# Supplementary material for: The transcription factor PAX5 activates human LINE1 retrotransposons to induce cellular senescence
Source: EMBO Rep. 2024 Jun 12;25(8):9. doi: 10.1038/s44319-024-00176-9 (PMC11315925; doi:10.1038/s44319-024-00176-9)
Supplement: Supplementary file 1 — Table EV1 [file 44319_2024_176_MOESM1_ESM.pdf]

Expanded View Tables

Table EV1

PAX5 mRNA expression in tissue from aging or HGPS individuals across different species

Species	Tissue	Log2 Fold Change	FDR
Human (Aging)	Lung	1.72	2.97e-02
Cynomolgus monkey (HGPS)	Skin	2.17	2.19e-02
	Lung	1.5	4.81e-02
Mouse (Aging)	Lung	2.17	1.57e-09
	Mesenteric adipose tissue	2.5	1.39e-05
	Gonadal adipose tissue	2.7	8.36e-05
	Liver	1.1	2.62e-03
	Muscle	4.6	1.81e-02
	Heart	1.4	4.26e-02

Table EV1 Alterations in PAX5 mRNA expression levels across diverse tissues from humans, mice, and Hutchinson-Gilford Progeria Syndrome (HGPS) cynomolgus monkeys from Aging Atlas.
