# Supplementary material for: The transcription factor PAX5 activates human LINE1 retrotransposons to induce cellular senescence
Source: EMBO Rep. 2024 Jun 12;25(8):9. doi: 10.1038/s44319-024-00176-9 (PMC11315925; doi:10.1038/s44319-024-00176-9)
Supplement: Supplementary file 2 — Appendix [file 44319_2024_176_MOESM2_ESM.pdf]

Appendix

The transcription factor PAX5 activates LINE1 retrotransposons to induce cellular senescence

Huanyin Tang[1, †], Jiaqing Yang[1, †], Junhao Xu[1], Weina Zhang[1], Anke Geng[1], Ying Jiang[2], Zhiyong Mao[1, *]

Table of content

Appendix Table S1 2

Appendix Figure S1 3

Appendix Figure S2 4

Appendix Figure S3 5

Appendix Table S1

Oligonucleotides used in this study. (F: forward; R: reverse)

ChIP-qPCR primers

Target	Sequence	Reference
LINE1	F, GTACCGGGTTCATCTCACTAGG<br>R, TGGTGCGCCGTTTCTTAAGC	
PAX5	F, CAACACACACACAGACGA<br>R, CTCTGTAAGTCCTGGTCT	
GAPDH*	F, CCAGGAGTGAGTGGAAGACAG<br>R, CTAGTTGCCTCCCCAAAGCA	Cecco *et al*, 2019

RT-qPCR primers

Target	Sequence	
LINE1	F, AGAGAGCAGTGGTTCTCCCA<br>R, CAGTCTGCCCGTTCTCAGAT	Cecco *et al*, 2019
PAX5	F, GAAGGTATTCAGGAGTCTCC<br>R, AGGCCATGGCTGAATACTCT	
IL1β	F, CGCCAGTGAAATGATGGCTTAT<br>R, CTGGAAGGAGCACTTCATCTGT	Cecco *et al*, 2019
IL6	F, CACTGGCAGAAAACAACCTGAA<br>R, ACCAGGCAAGTCTCCTCATTGA	Cecco *et al*, 2019
GAPDH	F, TTGAGGTCAATGAAGGGGTC<br>R, GAAGGTGAAGGTCGGAGTCA	Cecco *et al*, 2019

Appendix Figure S1

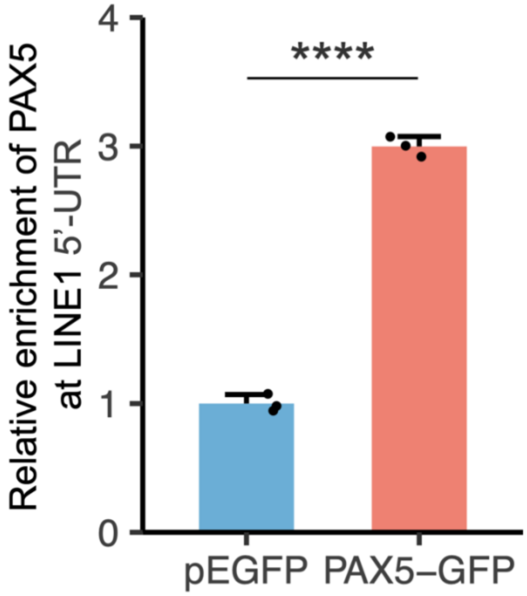

Appendix Figure S1

ChIP analysis displayed PAX5 enrichment at the designated LINE1 5'-UTR sites (n = 3). Error bars denote SD. Statistical significance was accessed by two-tailed Student's *t* test. ****$P <$ 0.0001.

Appendix Figure S2

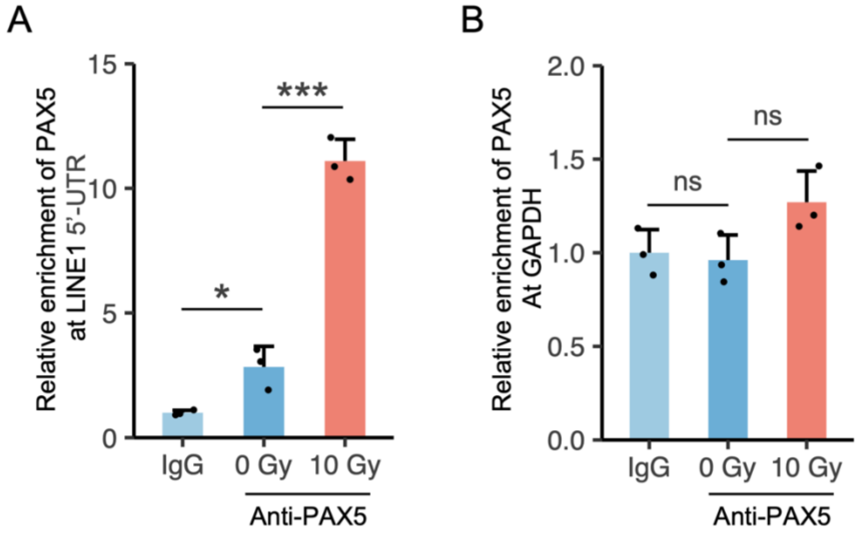

Appendix Figure S2

(A) ChIP analysis of PAX5 enrichment at the indicated LINE1 5'-UTR region in HCA2-hTERT cells collected on day 10 post 10 Gy X-ray irradiation (n = 3).

(B) ChIP analysis of PAX5 enrichment at negative control GAPDH in HCA2-hTERT cells collected on day 10 post 10 Gy X-ray irradiation (n = 3).

Error bars denote SD. Statistical significance was accessed by two-tailed Student's *t* test. ns, not significant; $*P < 0.05$; $***P < 0.001$.

Appendix Figure S3

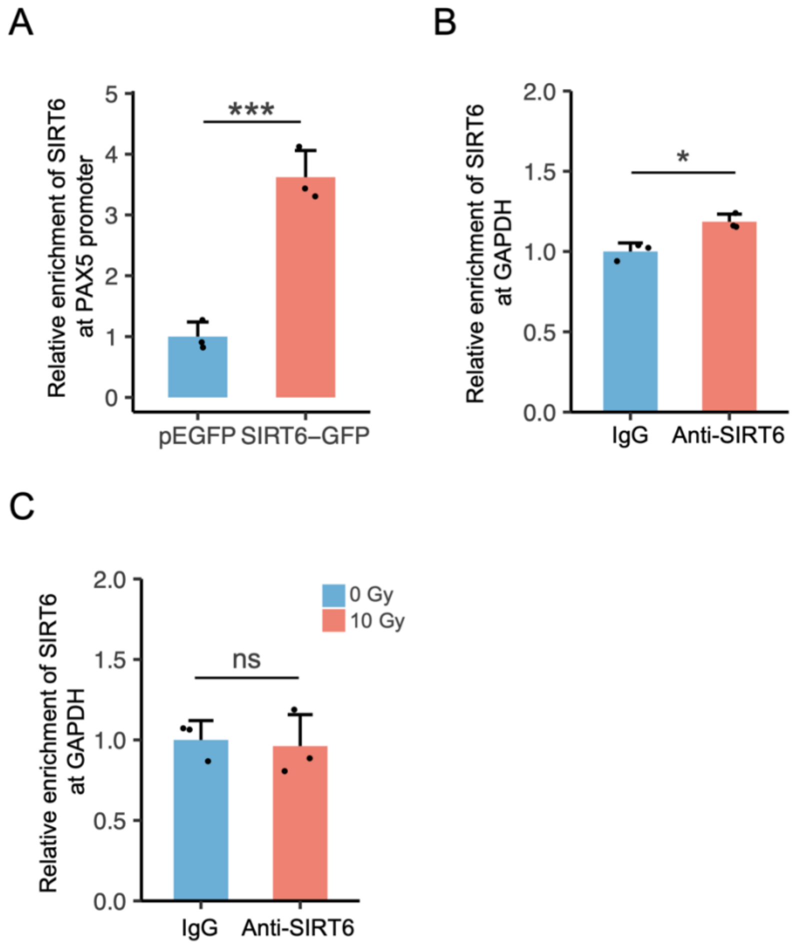

Appendix Figure S3

(A) ChIP analysis showed SIRT6 enrichment in PAX5 promoter in HEK293 cells.

(B) ChIP analysis showed endogenous SIRT6 at negative control GAPDH in HCA2-hTERT cells (n = 3).

(C) ChIP analysis of endogenous SIRT6 enrichment at negative control GAPDH in HCA2-hTERT cells collected on day 10 post 10 Gy X-ray irradiation (n = 3).

Error bars denote SD. Statistical significance was accessed by two-tailed Student's *t* test. ns, not significant; *$P < 0.05$; **$P < 0.01$; ***$P < 0.001$.
