# Supplementary figures and images for: The transcription factor PAX5 activates human LINE1 retrotransposons to induce cellular senescence
Source: EMBO Rep. 2024 Jun 12;25(8):9. doi: 10.1038/s44319-024-00176-9 (PMC11315925; doi:10.1038/s44319-024-00176-9)

Figure 2B

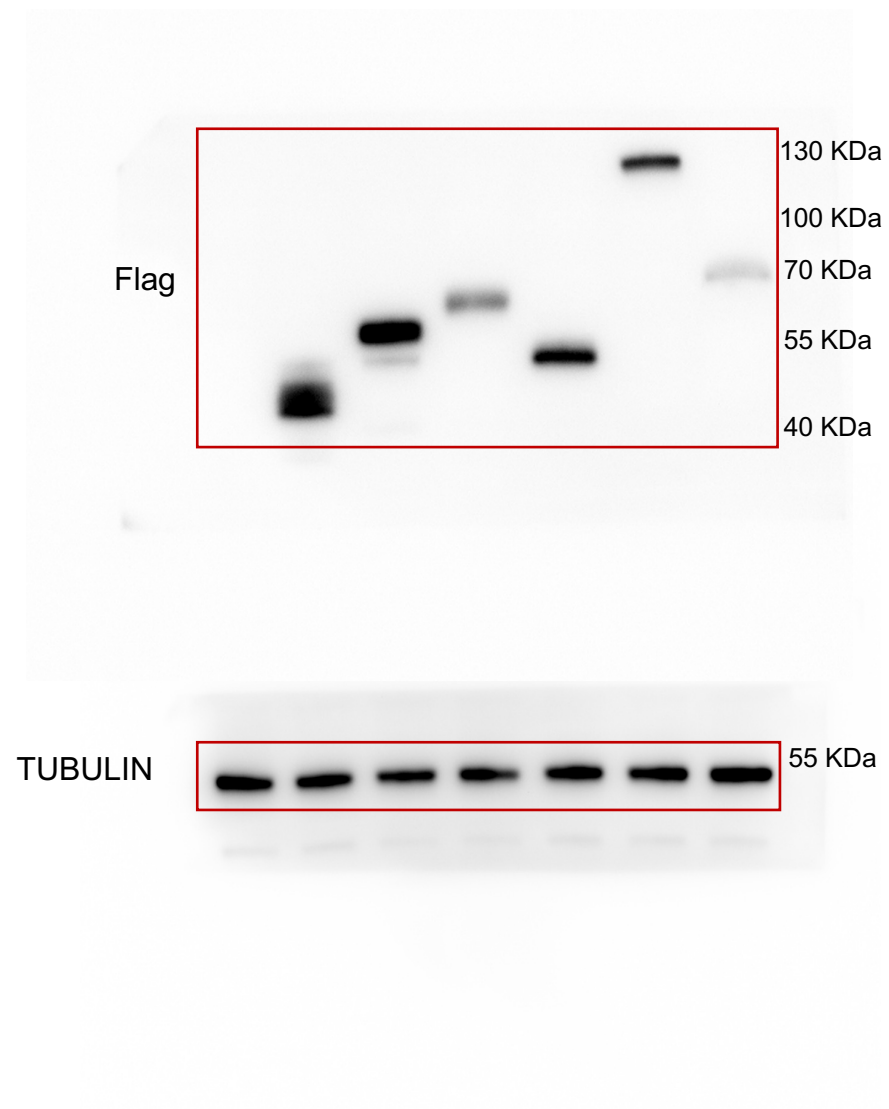

Supplement: Supplementary file 4 — Source data Fig. 2 [file 44319_2024_176_MOESM4_ESM.zip › SourceDara_ForFigure2/Figure2_cropping_area.pdf]

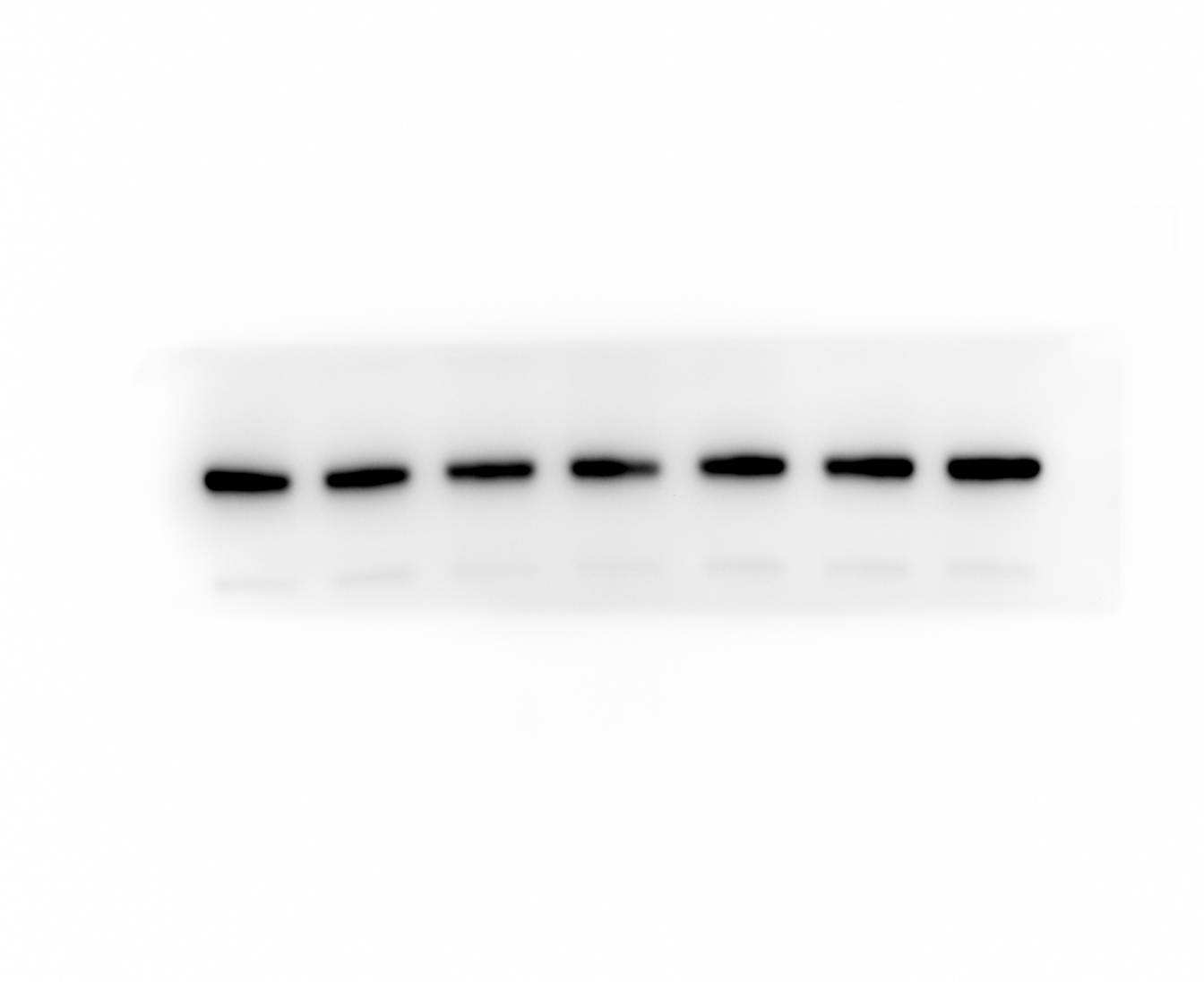

Supplement: Supplementary file 4 — Source data Fig. 2 [file 44319_2024_176_MOESM4_ESM.zip › SourceDara_ForFigure2/2B/western blot-TUBULIN.Tif]

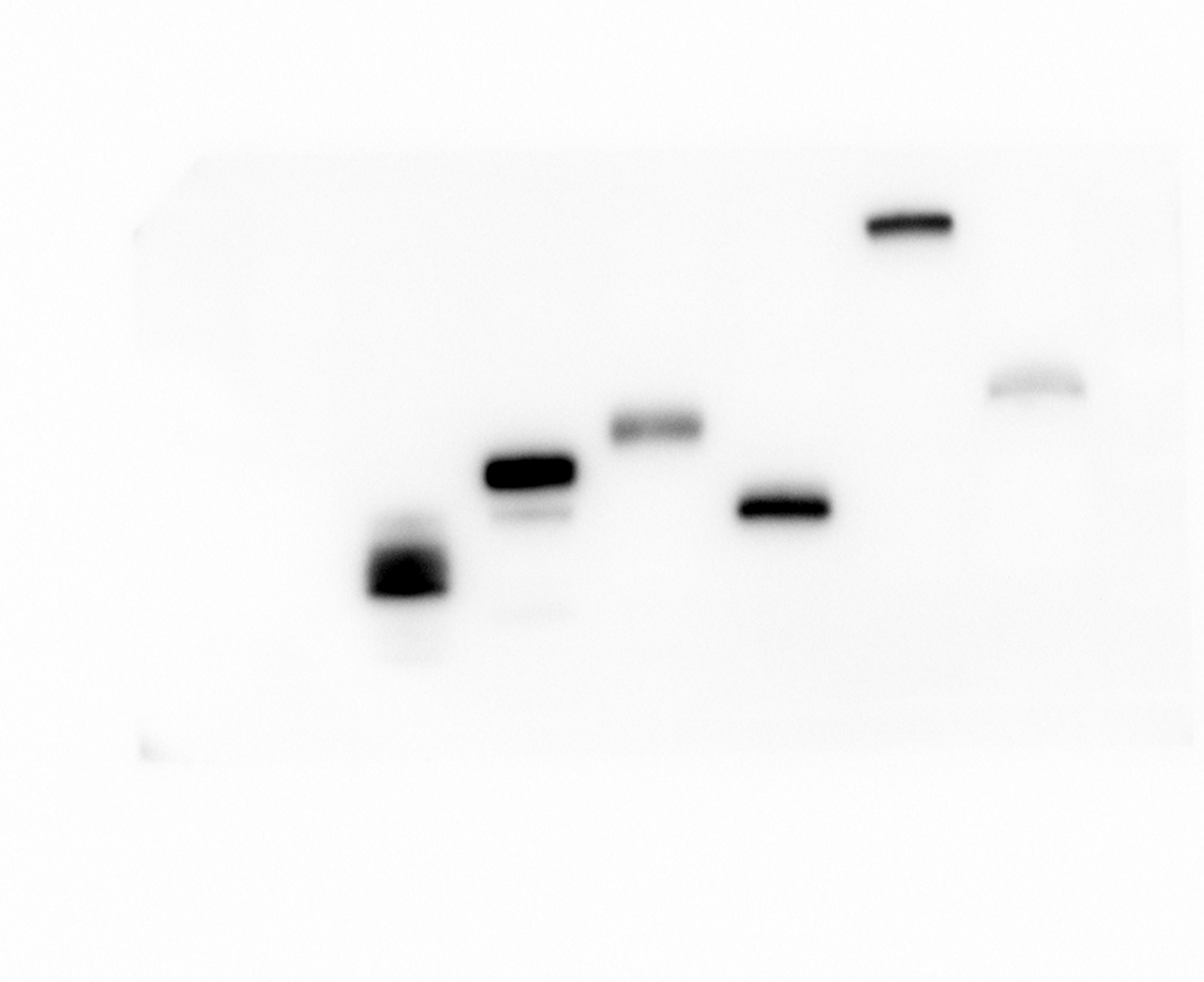

Supplement: Supplementary file 4 — Source data Fig. 2 [file 44319_2024_176_MOESM4_ESM.zip › SourceDara_ForFigure2/2B/western blot-FLAG.Tif]

Figure 3B

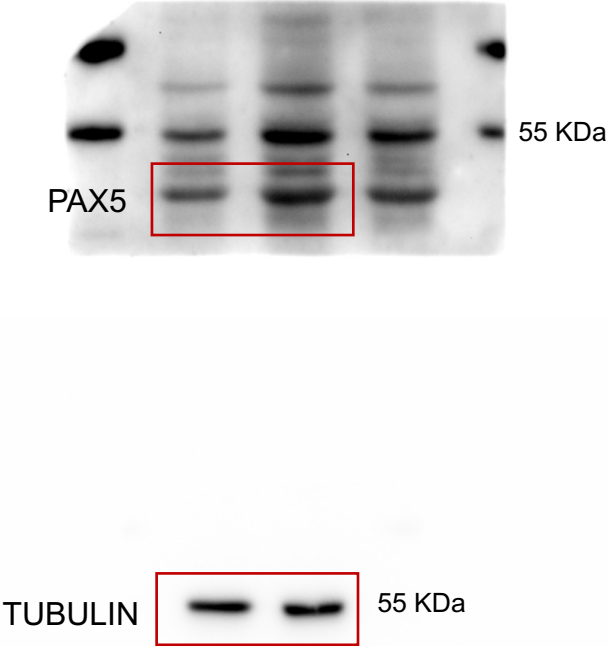

Figure 3C

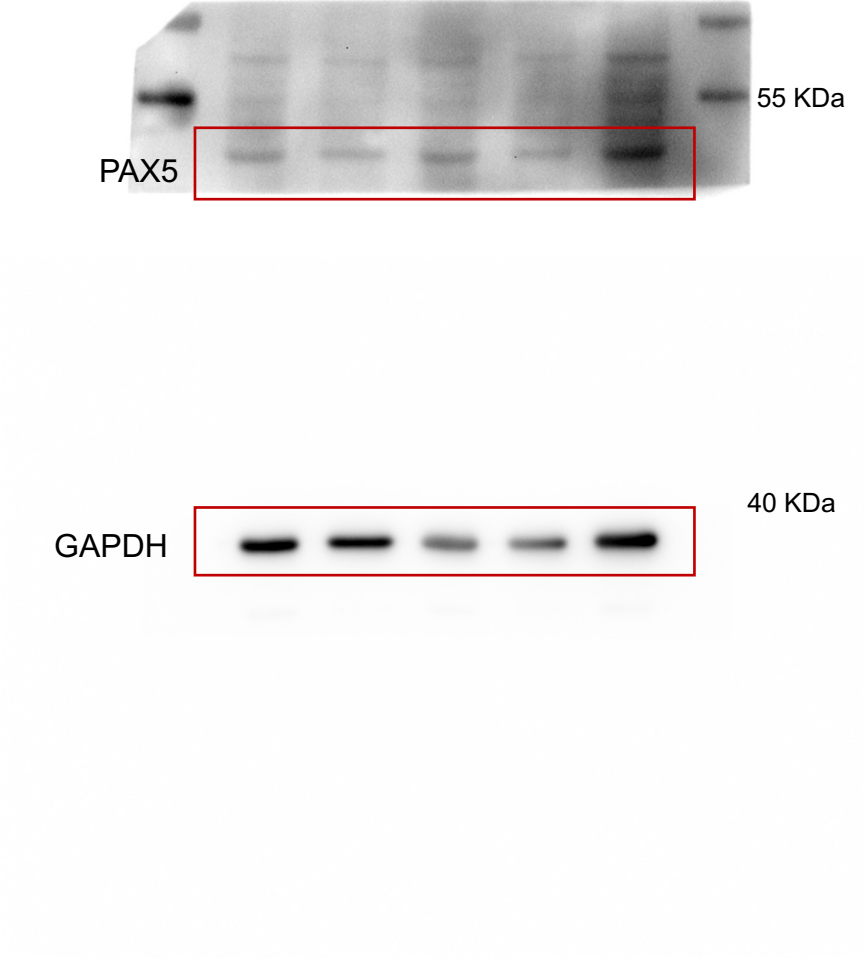

Figure 3D

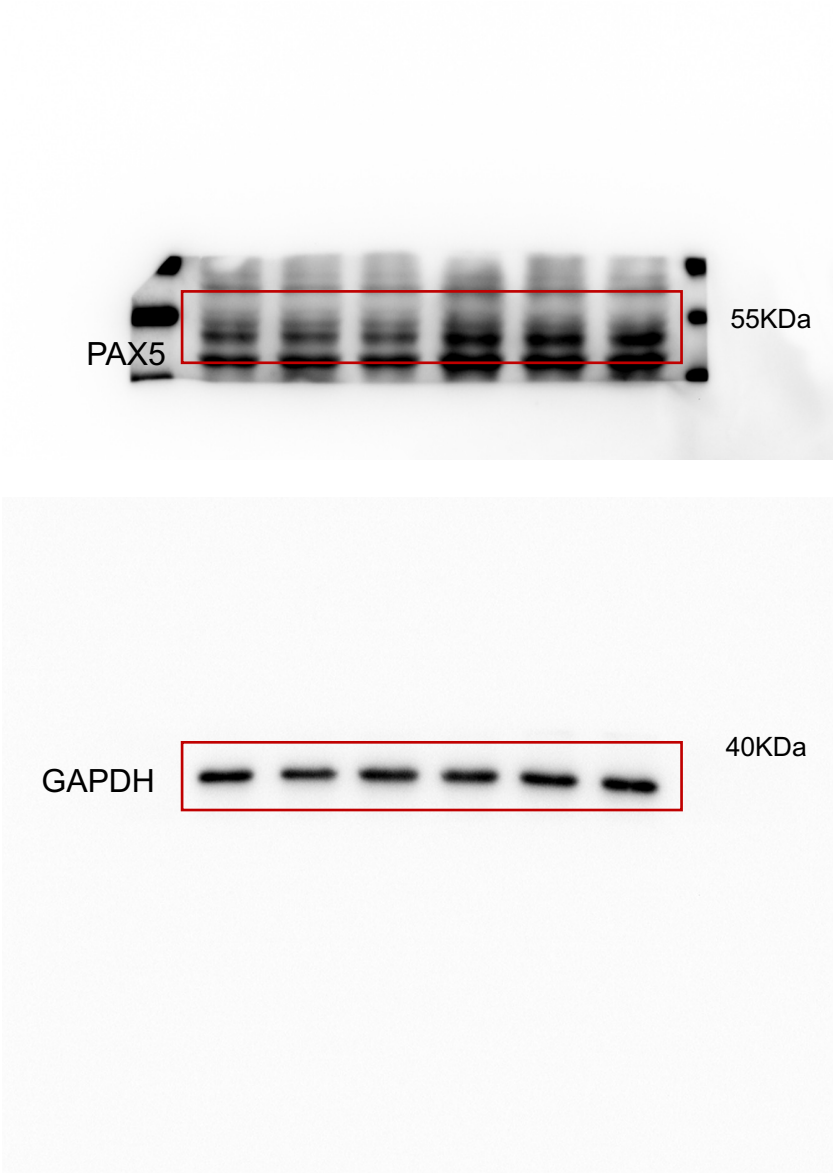

Figure 3G

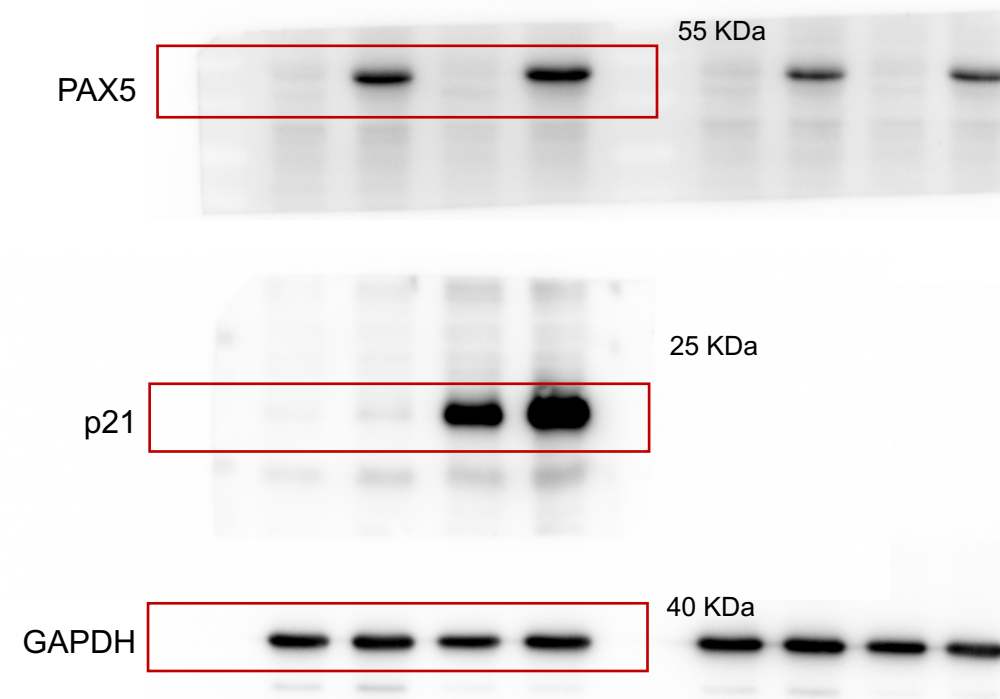

Supplement: Supplementary file 5 — Source data Fig. 3 [file 44319_2024_176_MOESM5_ESM.zip › SourceDara_ForFigure3/Figure3_cropping_area.pdf]

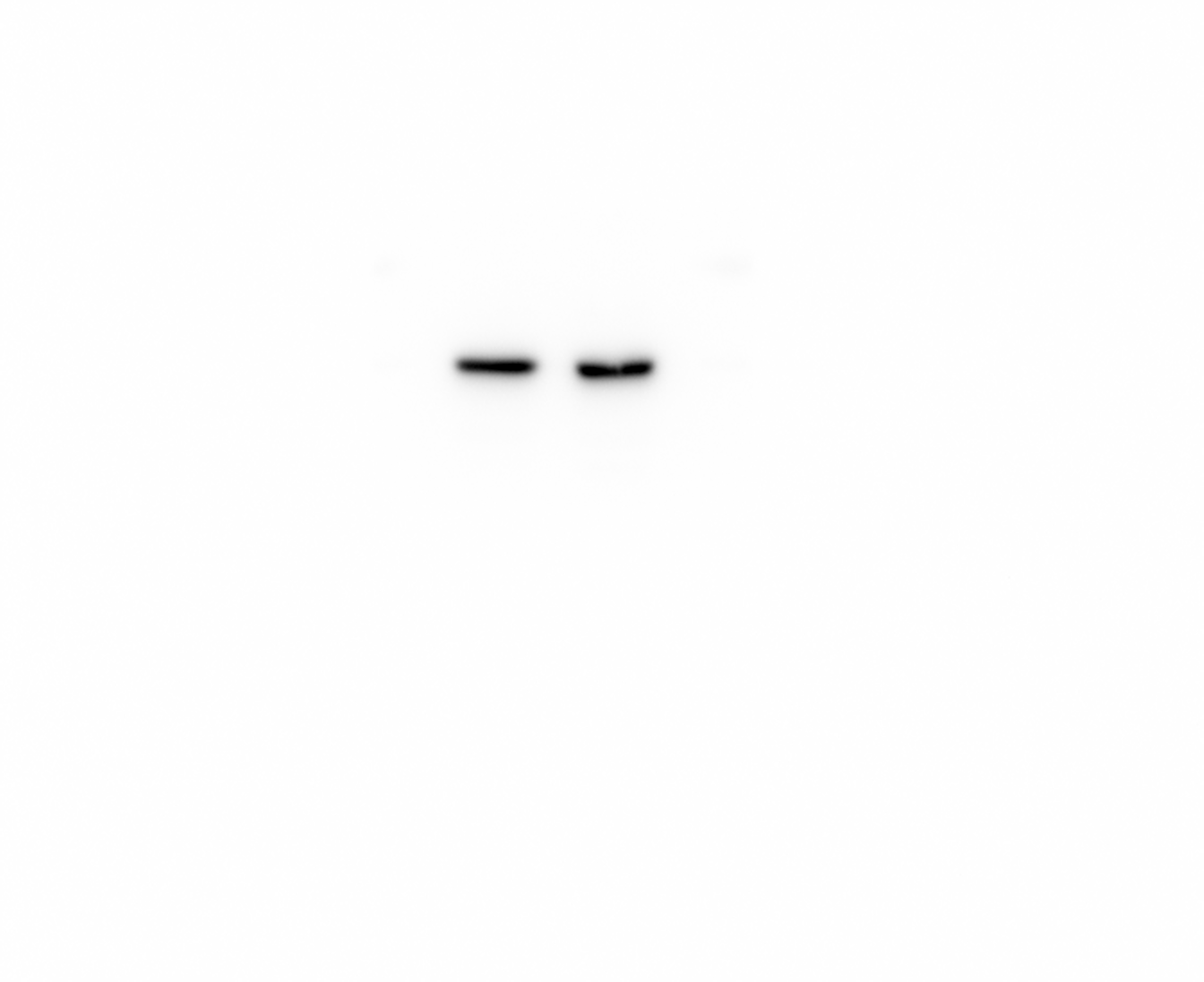

Supplement: Supplementary file 5 — Source data Fig. 3 [file 44319_2024_176_MOESM5_ESM.zip › SourceDara_ForFigure3/3B/western blot-TUBULIN.Tif]

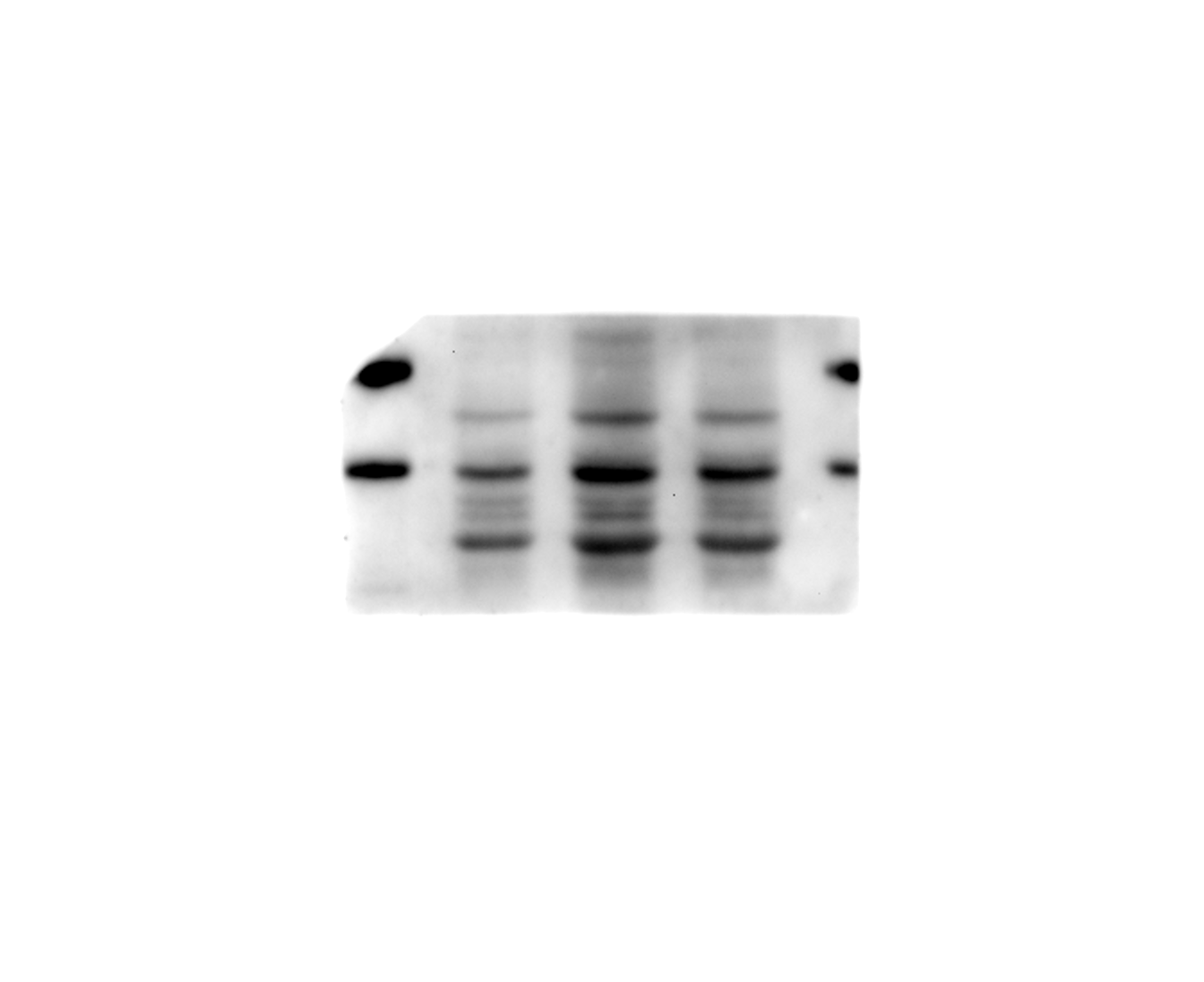

Supplement: Supplementary file 5 — Source data Fig. 3 [file 44319_2024_176_MOESM5_ESM.zip › SourceDara_ForFigure3/3B/western blot-PAX5.Tif]

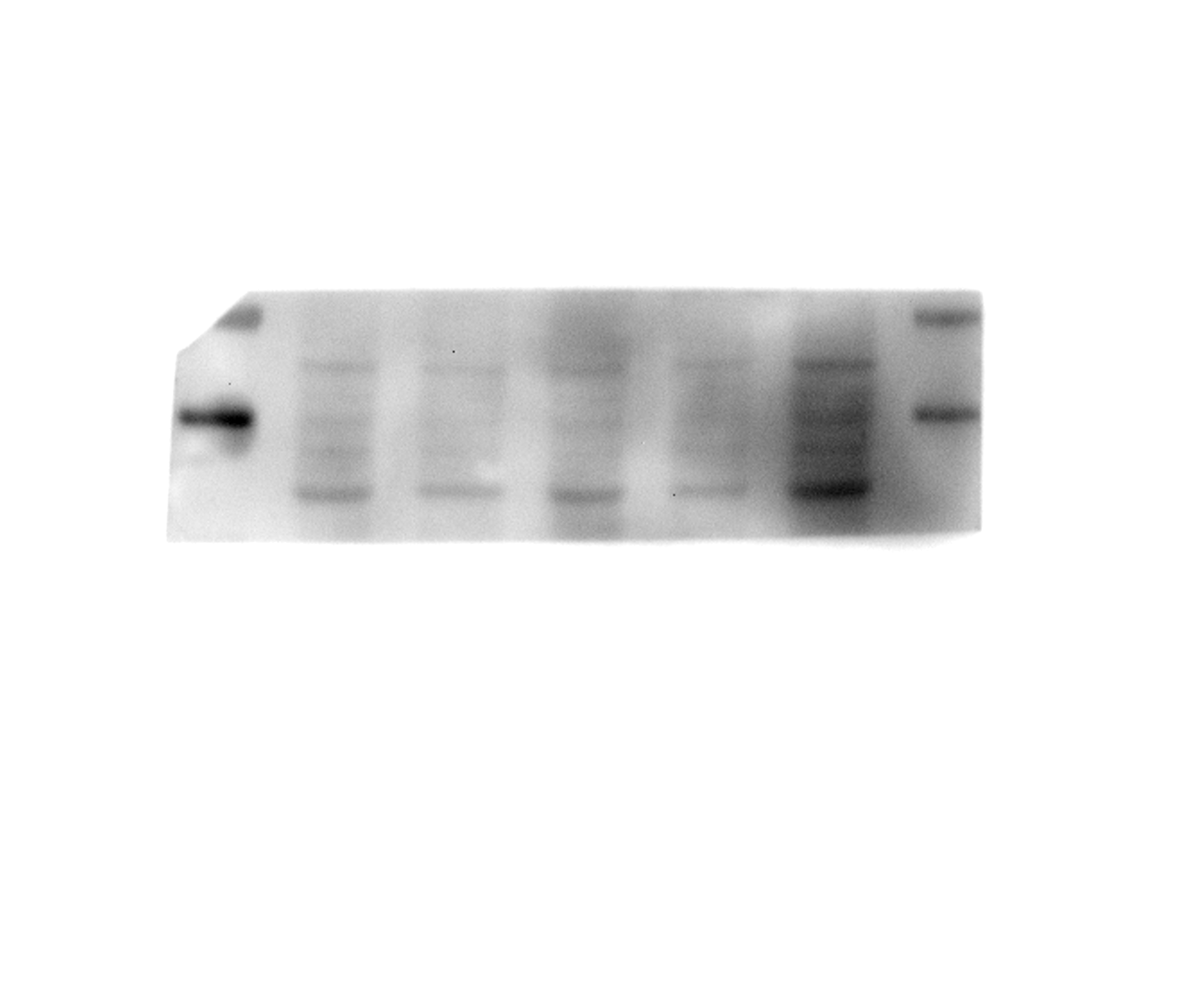

Supplement: Supplementary file 5 — Source data Fig. 3 [file 44319_2024_176_MOESM5_ESM.zip › SourceDara_ForFigure3/3C/western blot-PAX5.Tif]

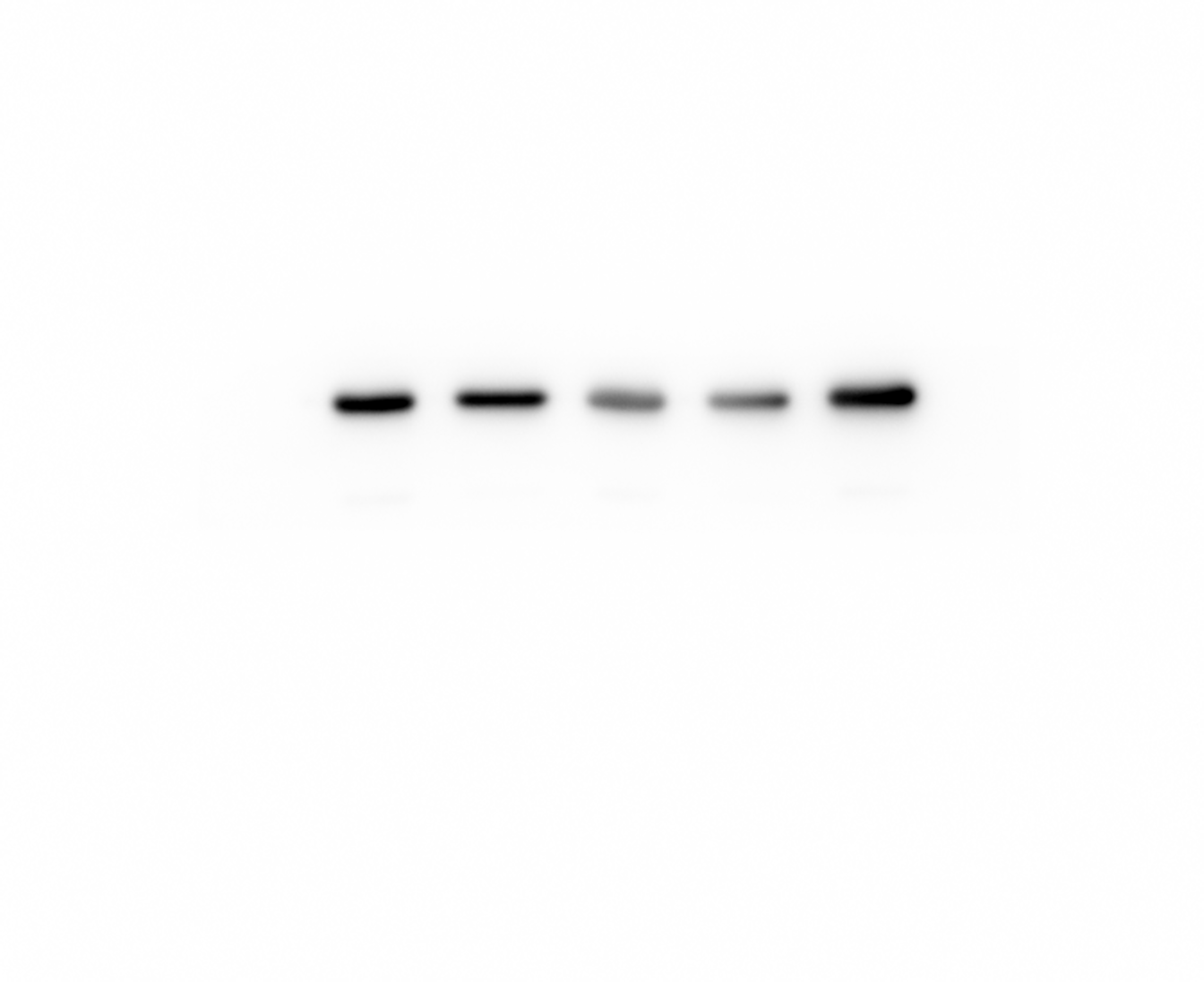

Supplement: Supplementary file 5 — Source data Fig. 3 [file 44319_2024_176_MOESM5_ESM.zip › SourceDara_ForFigure3/3C/western blot-GAPDH.Tif]

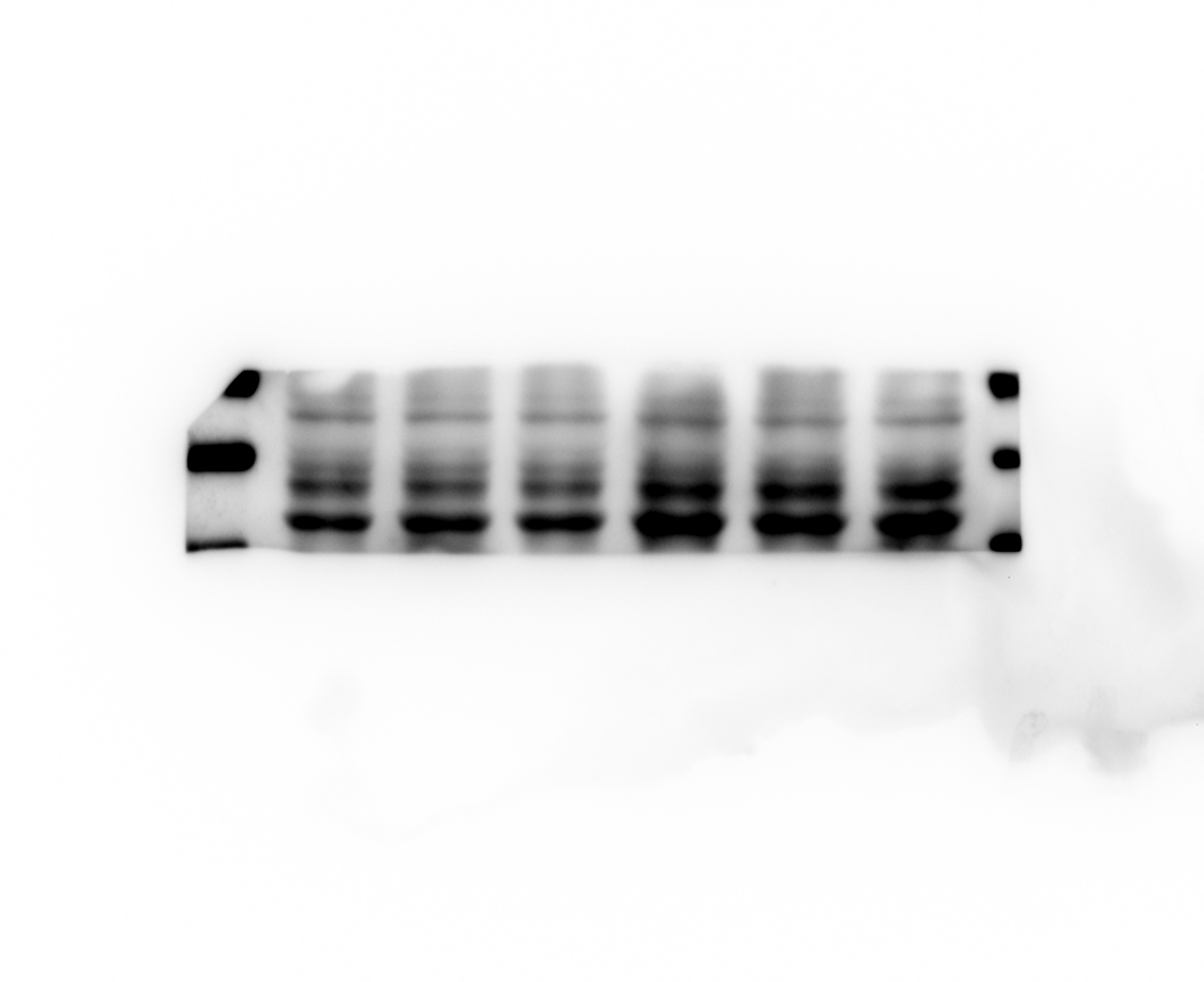

Supplement: Supplementary file 5 — Source data Fig. 3 [file 44319_2024_176_MOESM5_ESM.zip › SourceDara_ForFigure3/3D/western blot-PAX5.Tif]

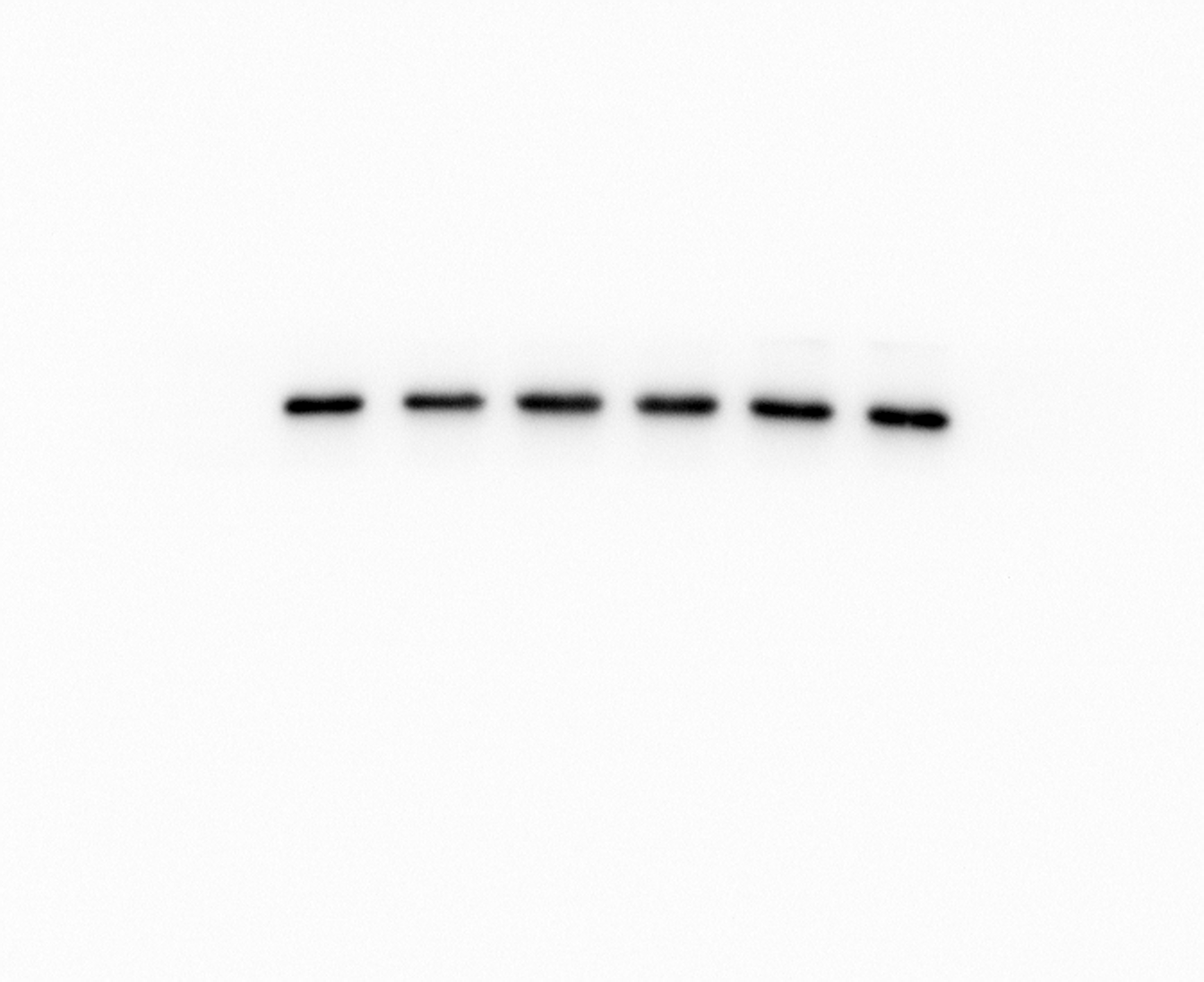

Supplement: Supplementary file 5 — Source data Fig. 3 [file 44319_2024_176_MOESM5_ESM.zip › SourceDara_ForFigure3/3D/western blot-GAPDH.Tif]

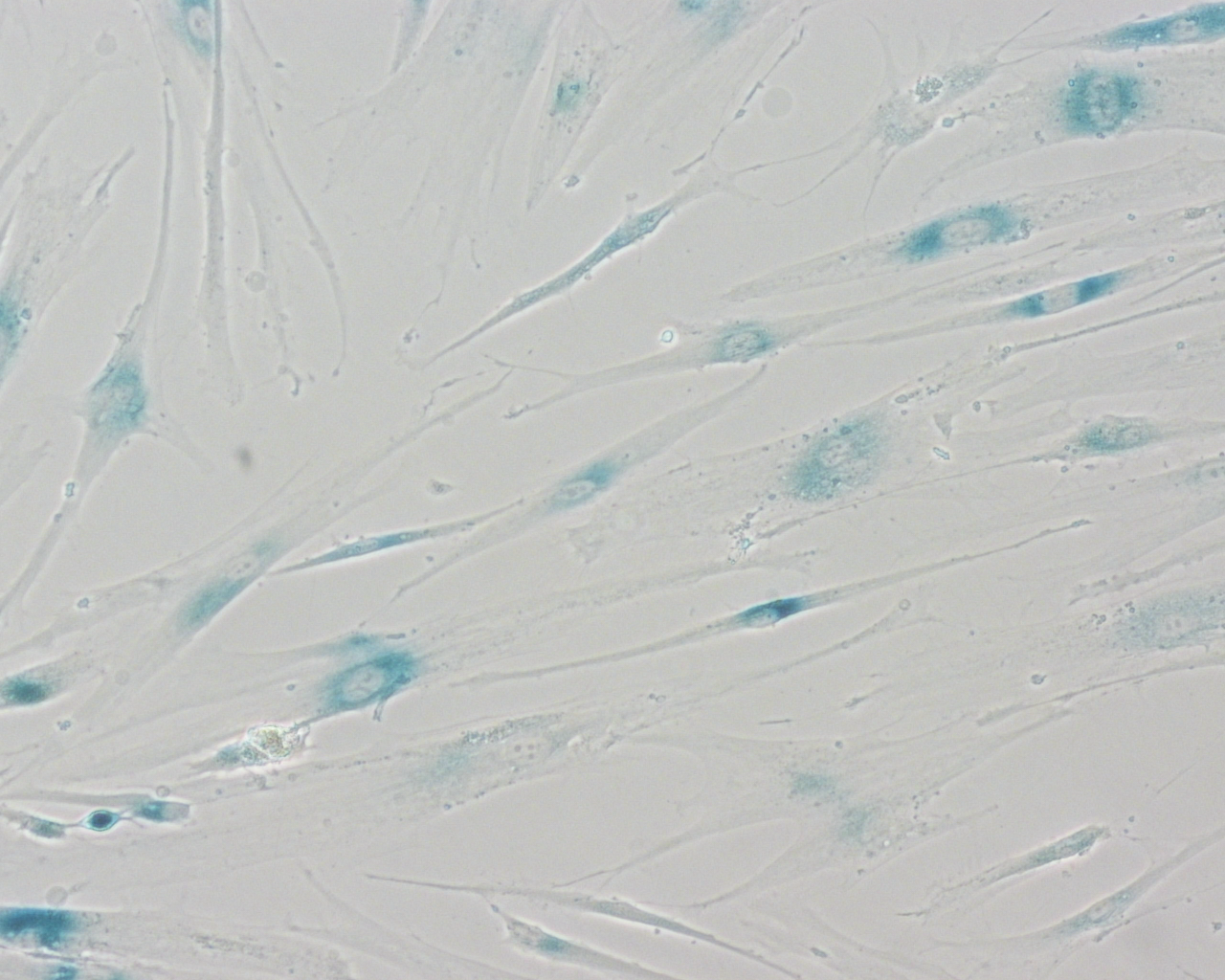

Supplement: Supplementary file 5 — Source data Fig. 3 [file 44319_2024_176_MOESM5_ESM.zip › SourceDara_ForFigure3/3J/pControl-DMSO.tif]

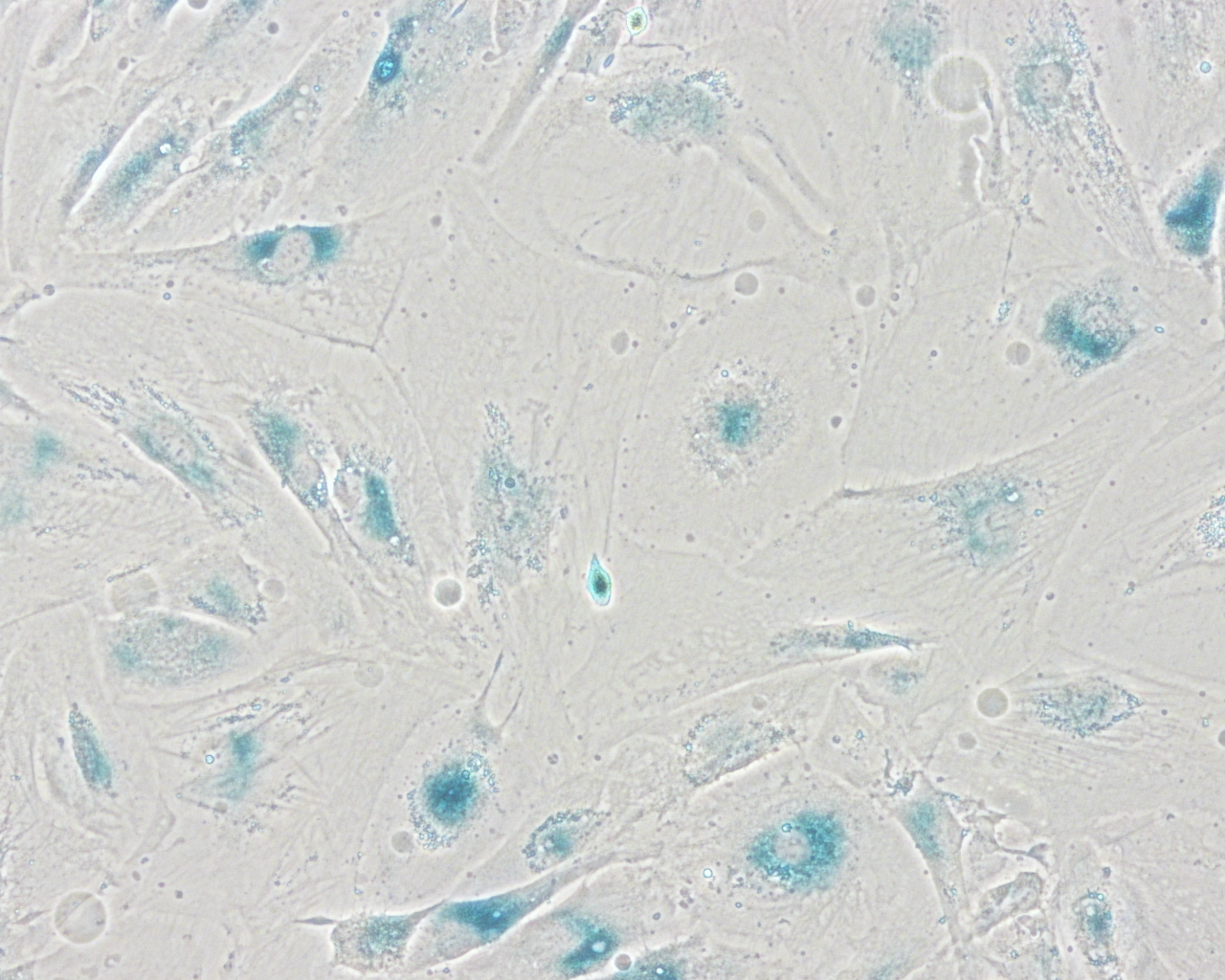

Supplement: Supplementary file 5 — Source data Fig. 3 [file 44319_2024_176_MOESM5_ESM.zip › SourceDara_ForFigure3/3J/pPAX5-3TC.tif]

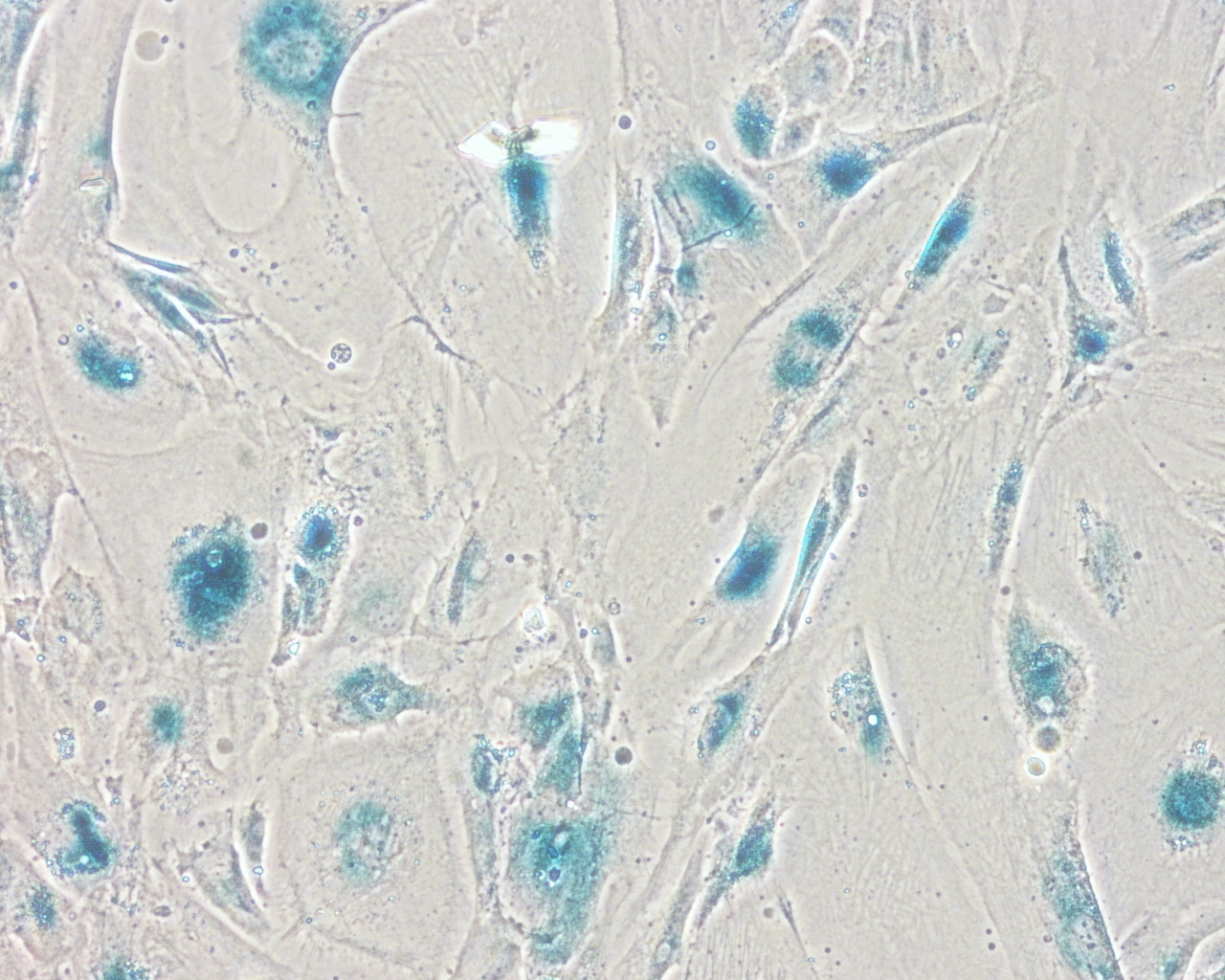

Supplement: Supplementary file 5 — Source data Fig. 3 [file 44319_2024_176_MOESM5_ESM.zip › SourceDara_ForFigure3/3J/pPAX5-DMSO.tif]

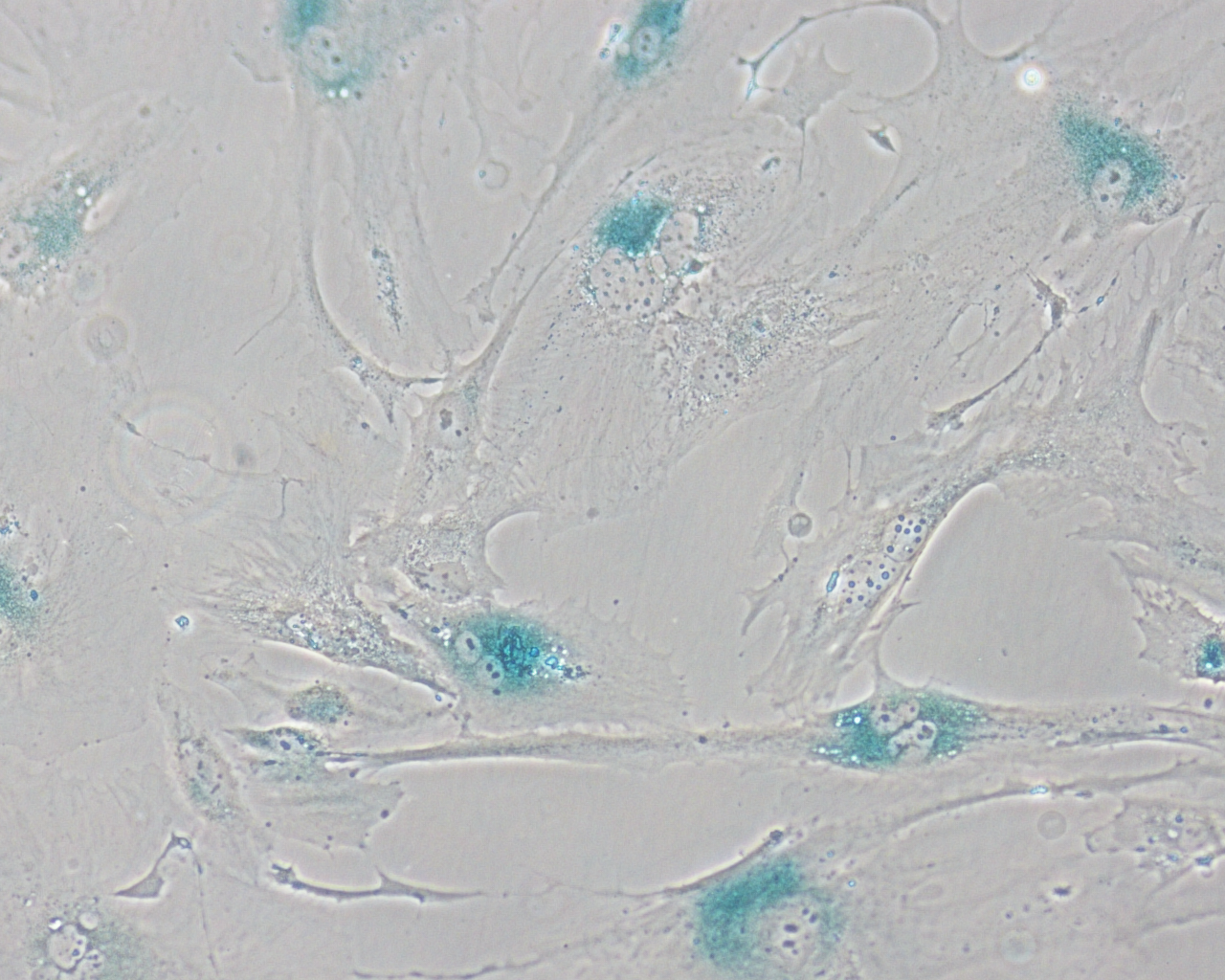

Supplement: Supplementary file 5 — Source data Fig. 3 [file 44319_2024_176_MOESM5_ESM.zip › SourceDara_ForFigure3/3H/pPAX5-10Gy.tif]

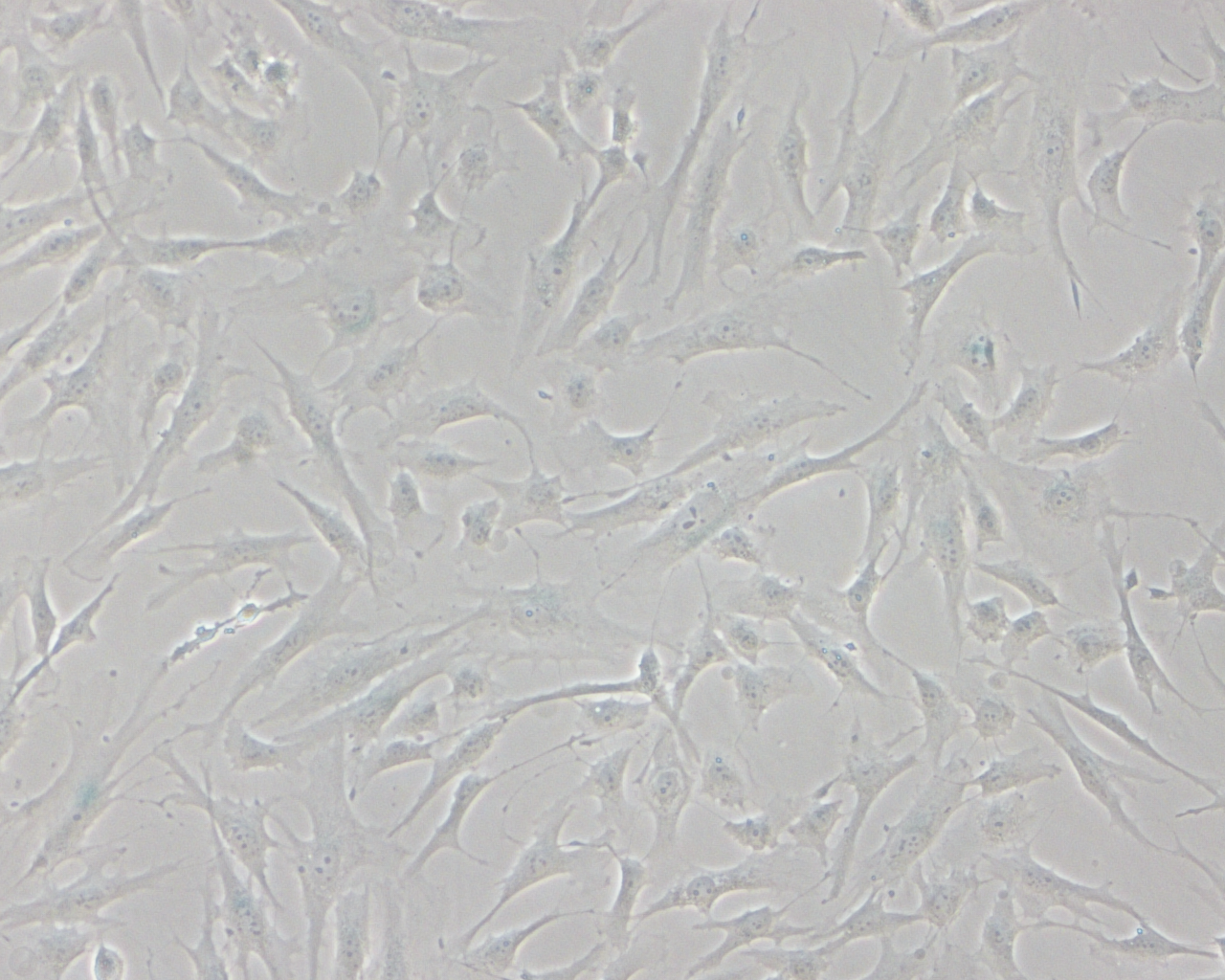

Supplement: Supplementary file 5 — Source data Fig. 3 [file 44319_2024_176_MOESM5_ESM.zip › SourceDara_ForFigure3/3H/pControl-0Gy.tif]

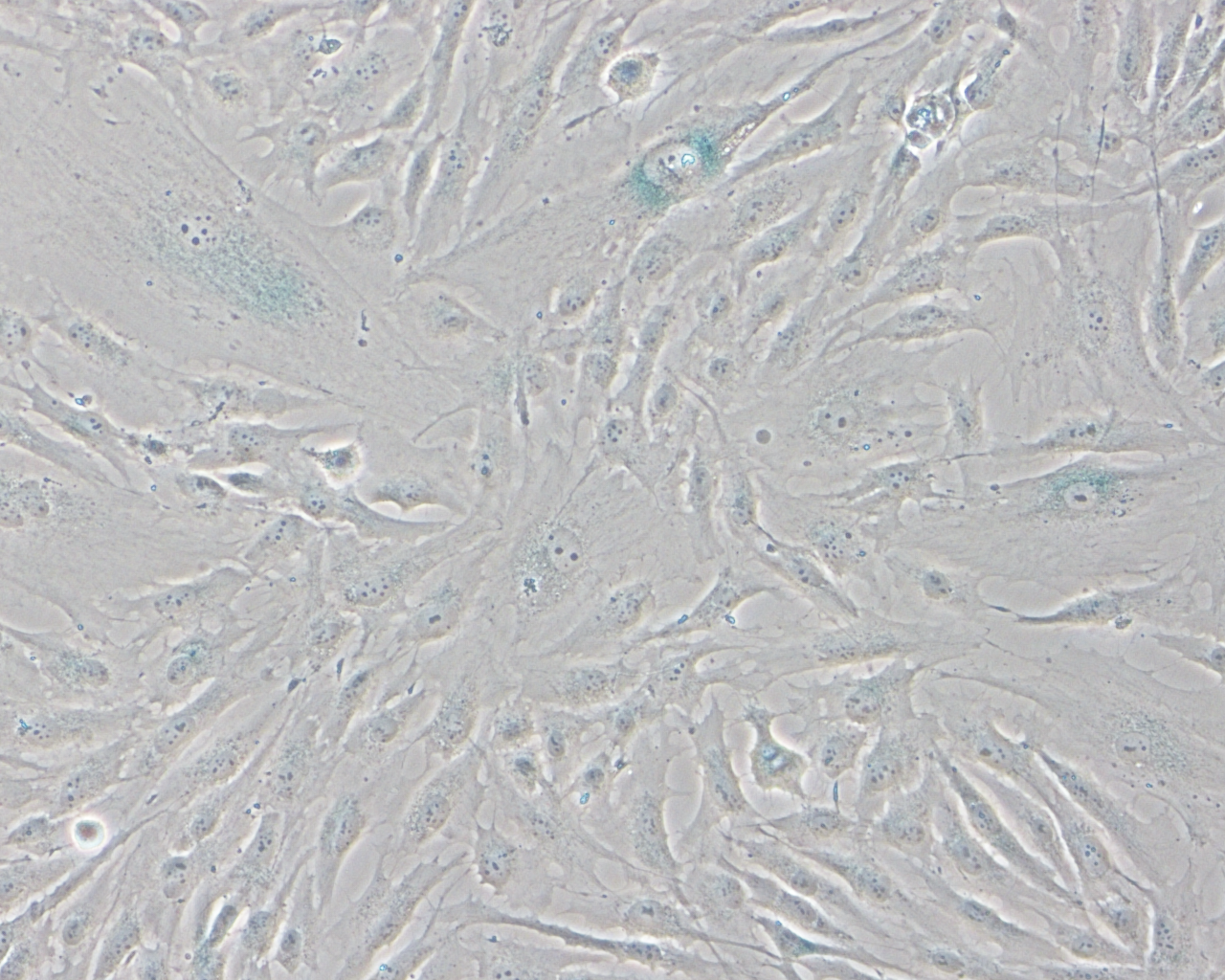

Supplement: Supplementary file 5 — Source data Fig. 3 [file 44319_2024_176_MOESM5_ESM.zip › SourceDara_ForFigure3/3H/pPAX5-0Gy.tif]

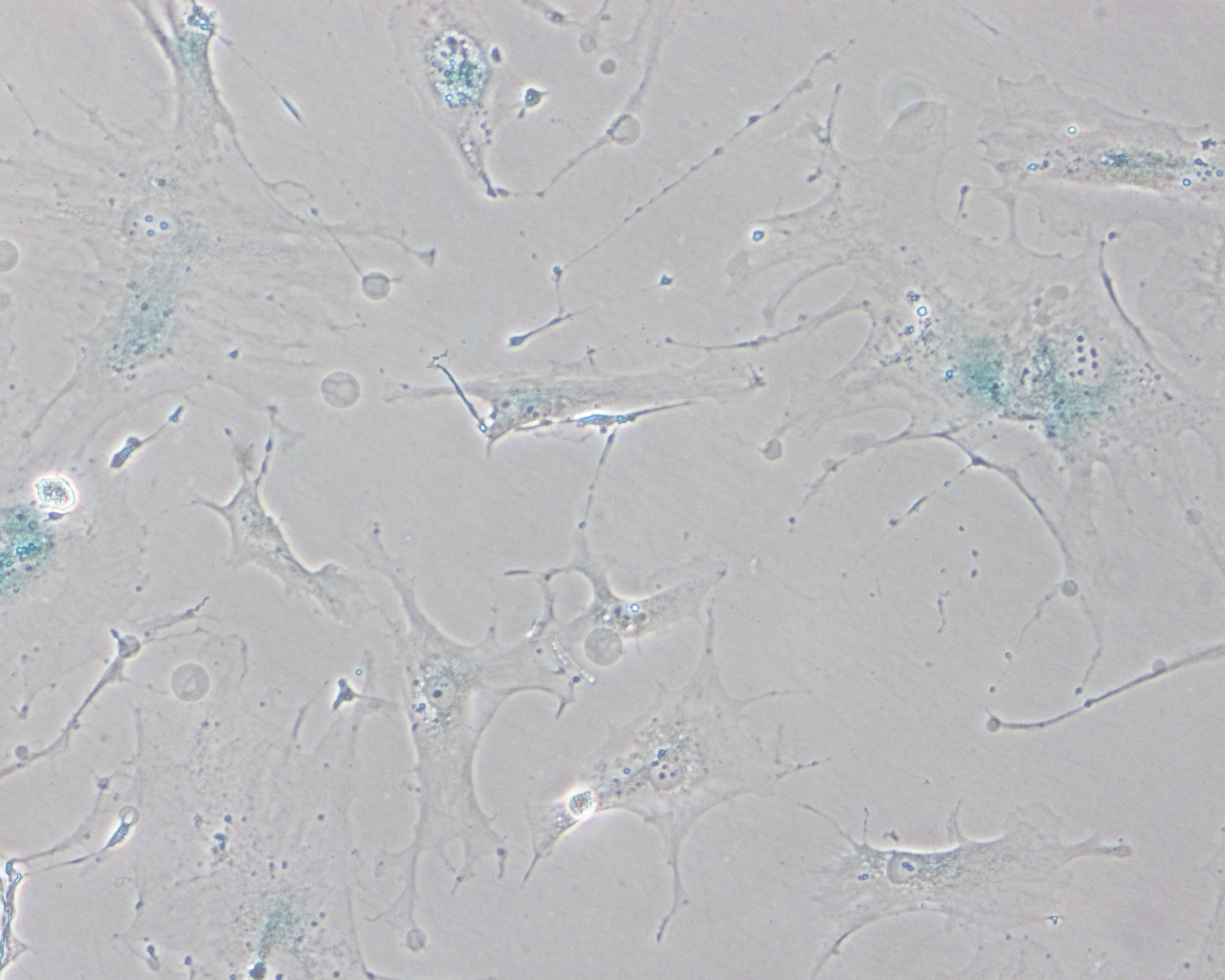

Supplement: Supplementary file 5 — Source data Fig. 3 [file 44319_2024_176_MOESM5_ESM.zip › SourceDara_ForFigure3/3H/pControl-10Gy.tif]

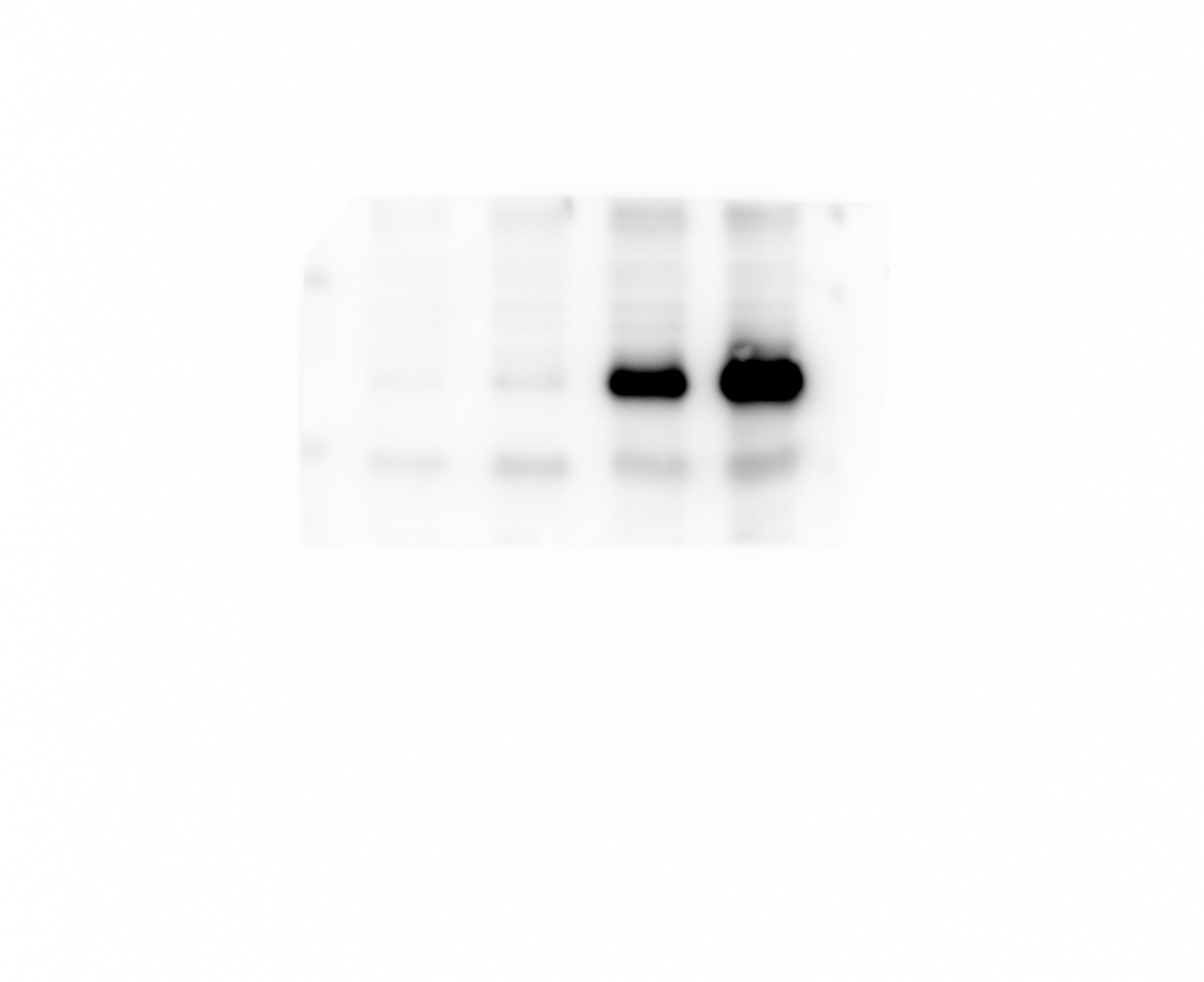

Supplement: Supplementary file 5 — Source data Fig. 3 [file 44319_2024_176_MOESM5_ESM.zip › SourceDara_ForFigure3/3G/western blot-p21.Tif]

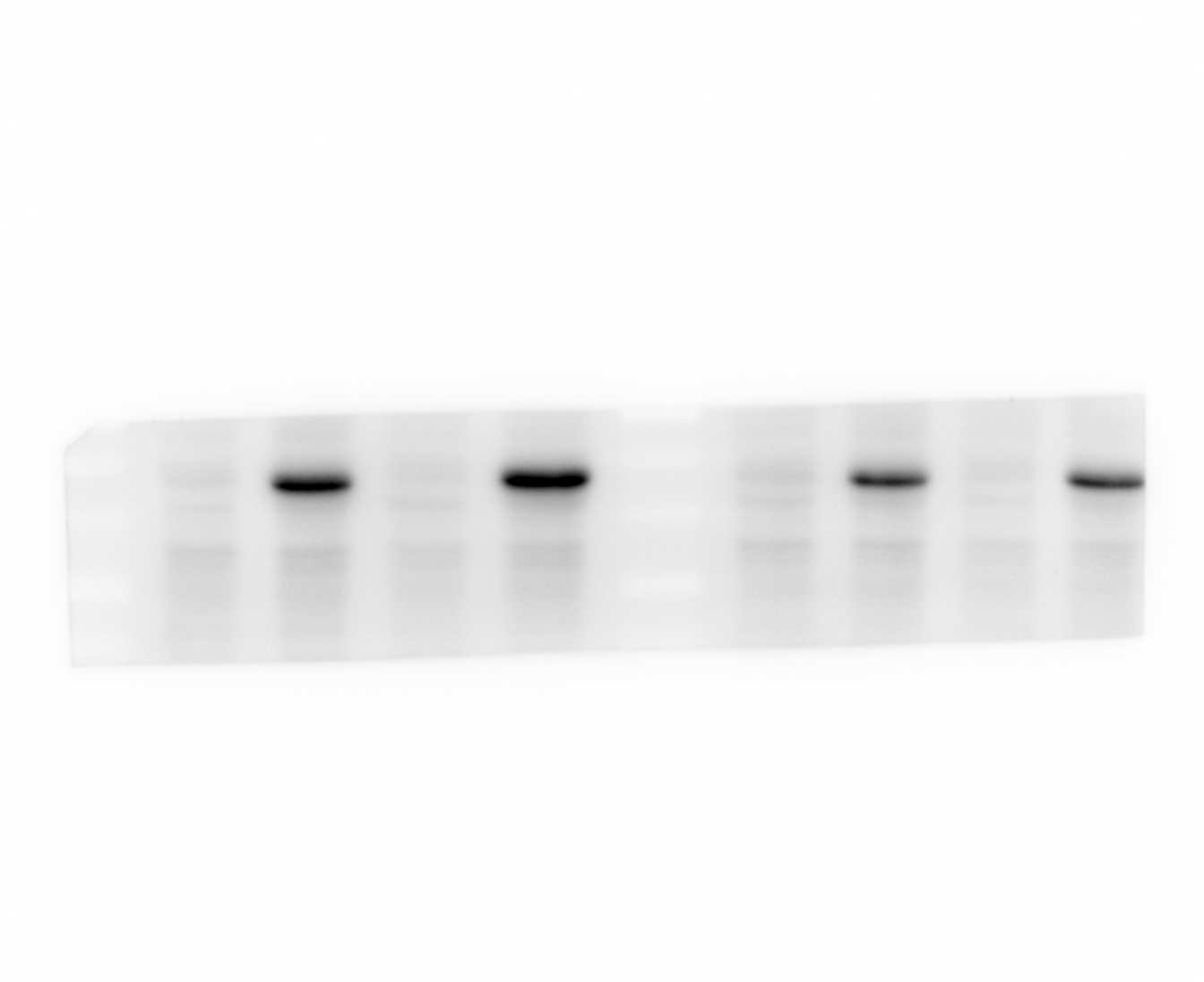

Supplement: Supplementary file 5 — Source data Fig. 3 [file 44319_2024_176_MOESM5_ESM.zip › SourceDara_ForFigure3/3G/western blot-PAX5.Tif]

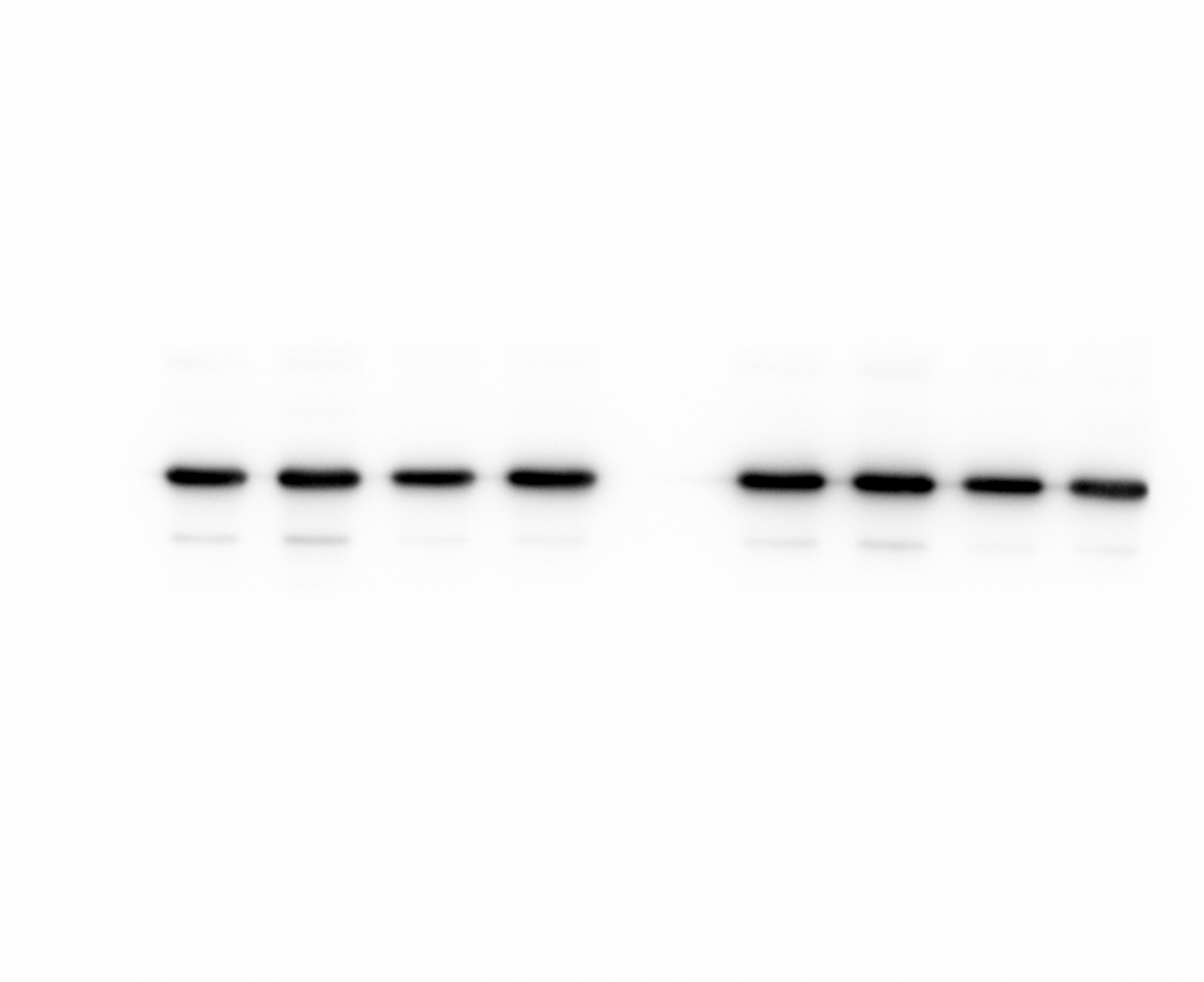

Supplement: Supplementary file 5 — Source data Fig. 3 [file 44319_2024_176_MOESM5_ESM.zip › SourceDara_ForFigure3/3G/western blot-GAPDH.Tif]

Figure 4A

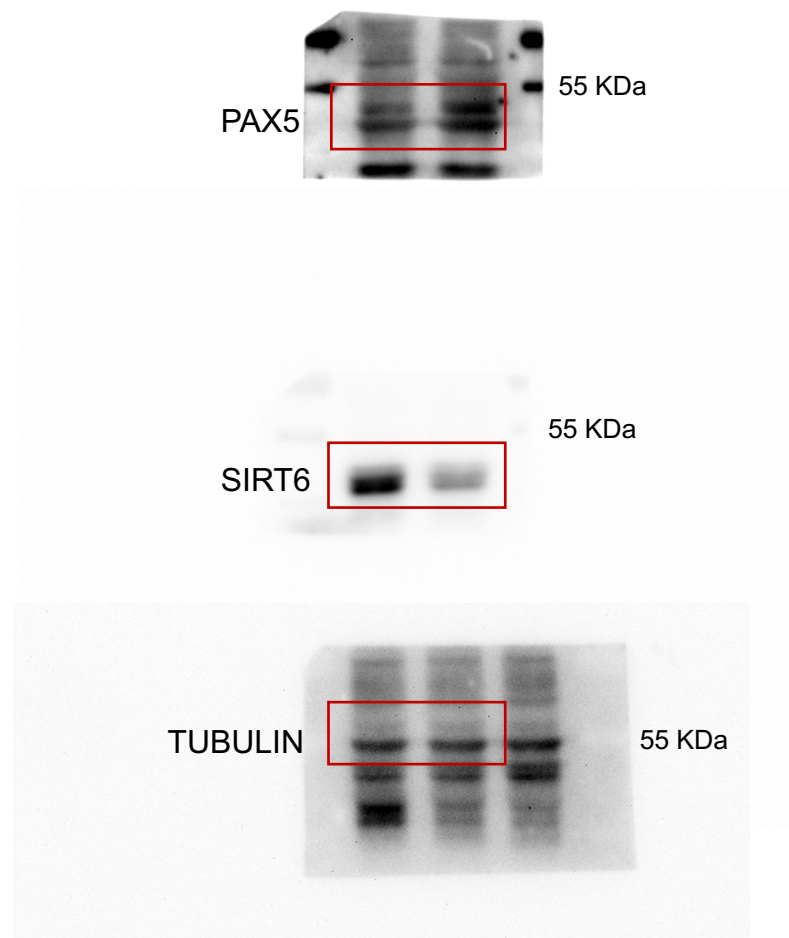

Supplement: Supplementary file 6 — Source data Fig. 4 [file 44319_2024_176_MOESM6_ESM.zip › SourceDara_ForFigure4/Figure4_cropping_area.pdf]

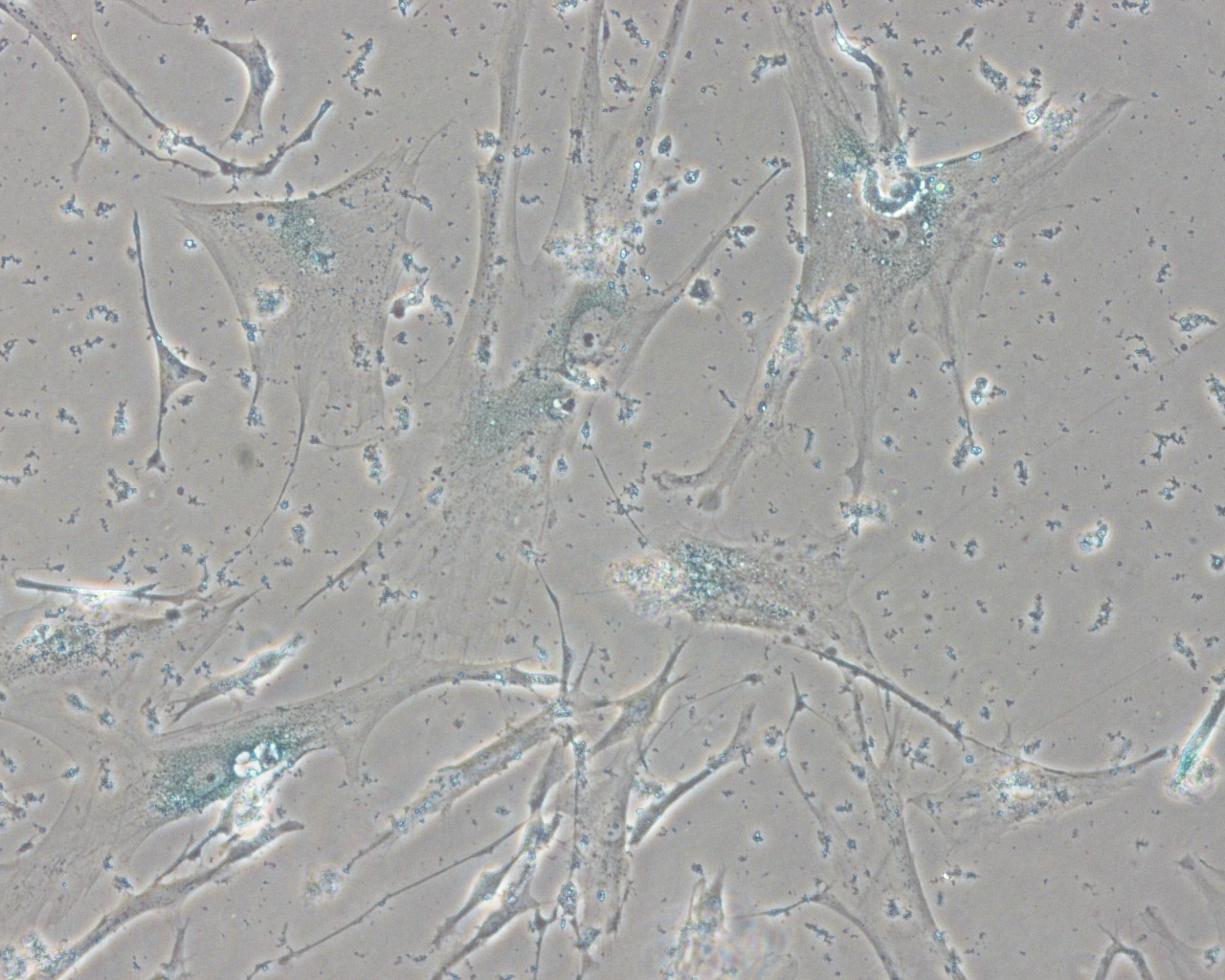

Supplement: Supplementary file 6 — Source data Fig. 4 [file 44319_2024_176_MOESM6_ESM.zip › SourceDara_ForFigure4/4K/pSIRT6-10Gy.tif]

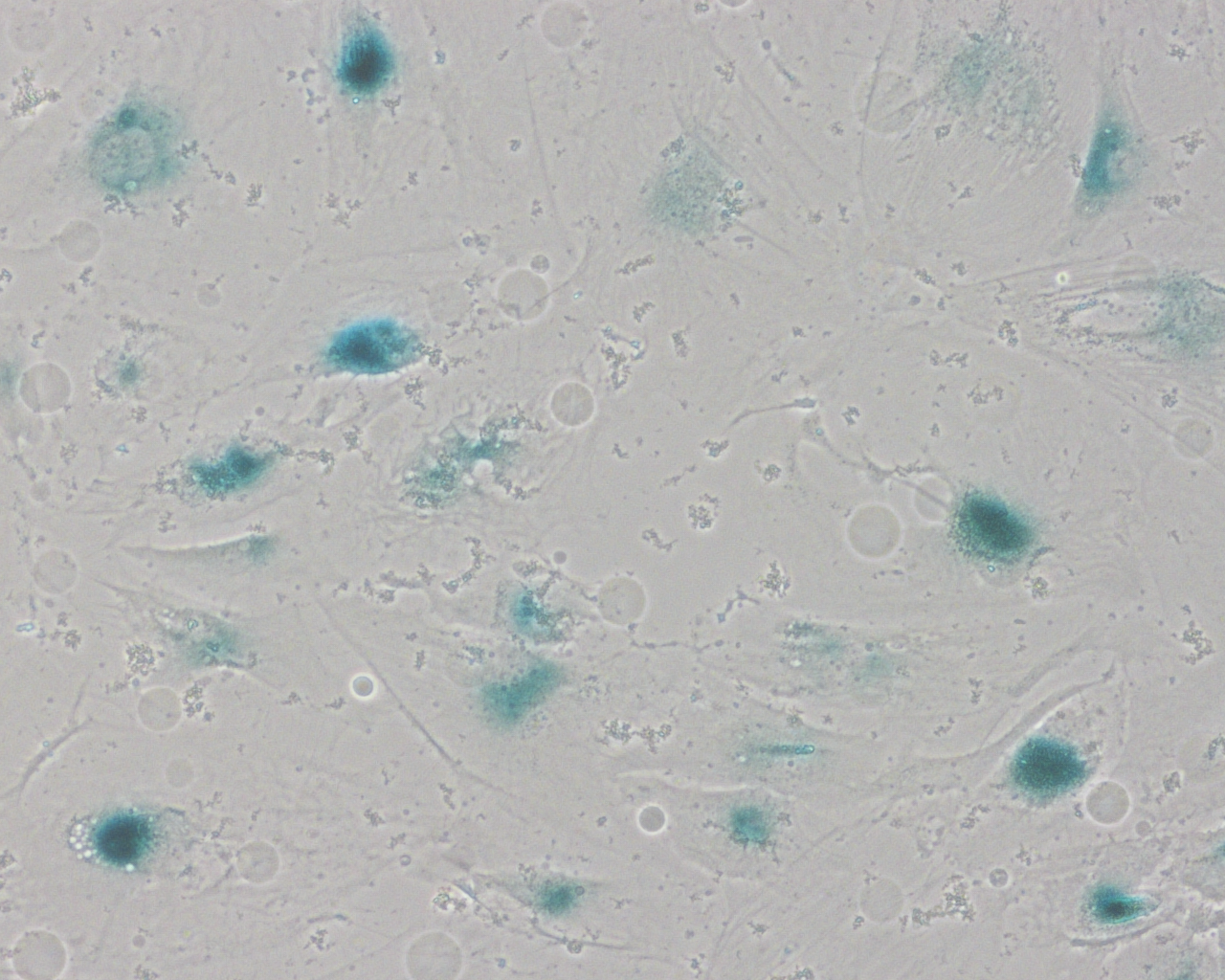

Supplement: Supplementary file 6 — Source data Fig. 4 [file 44319_2024_176_MOESM6_ESM.zip › SourceDara_ForFigure4/4K/pPAX5-10Gy.tif]

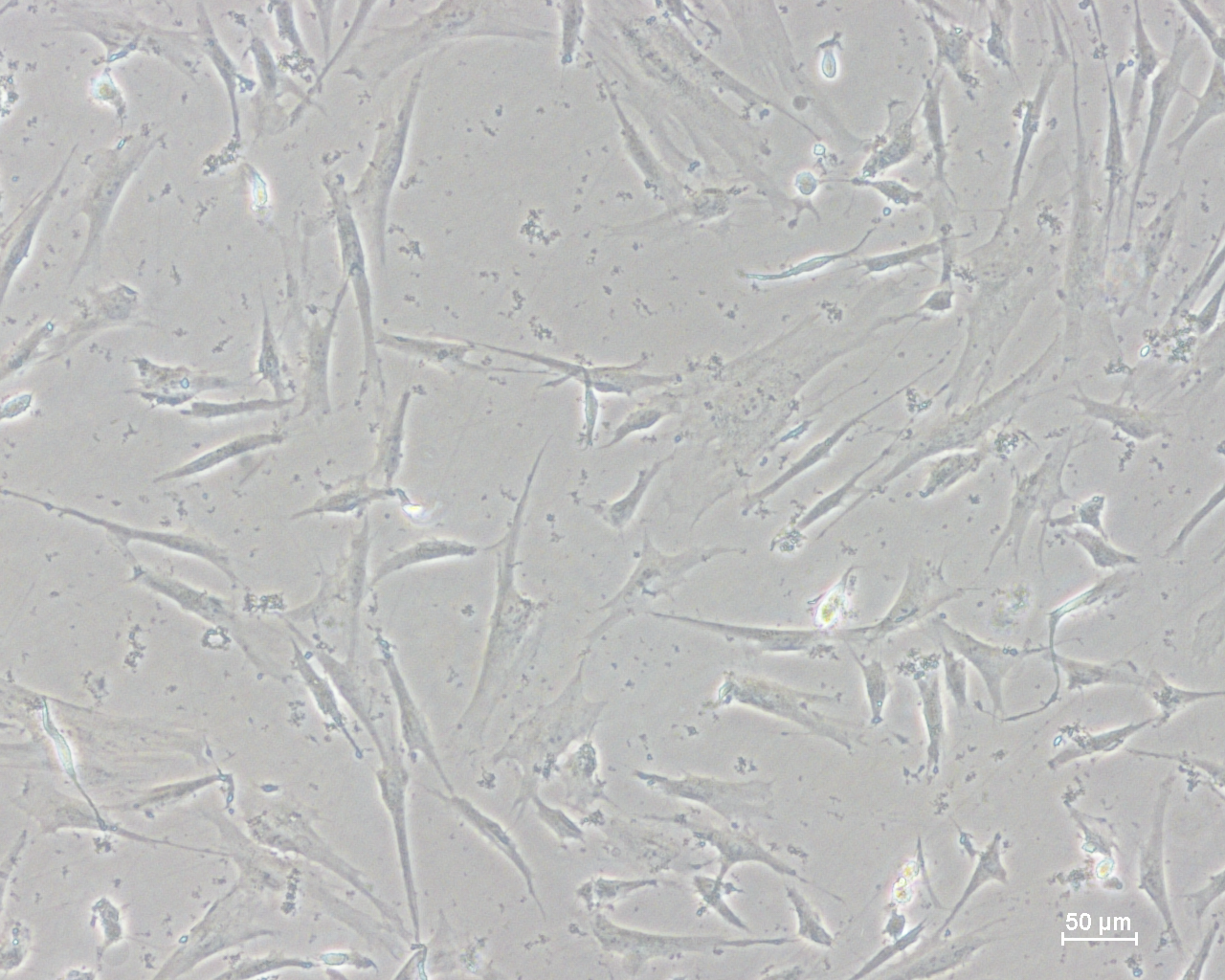

Supplement: Supplementary file 6 — Source data Fig. 4 [file 44319_2024_176_MOESM6_ESM.zip › SourceDara_ForFigure4/4K/pControl-0Gy.tif]

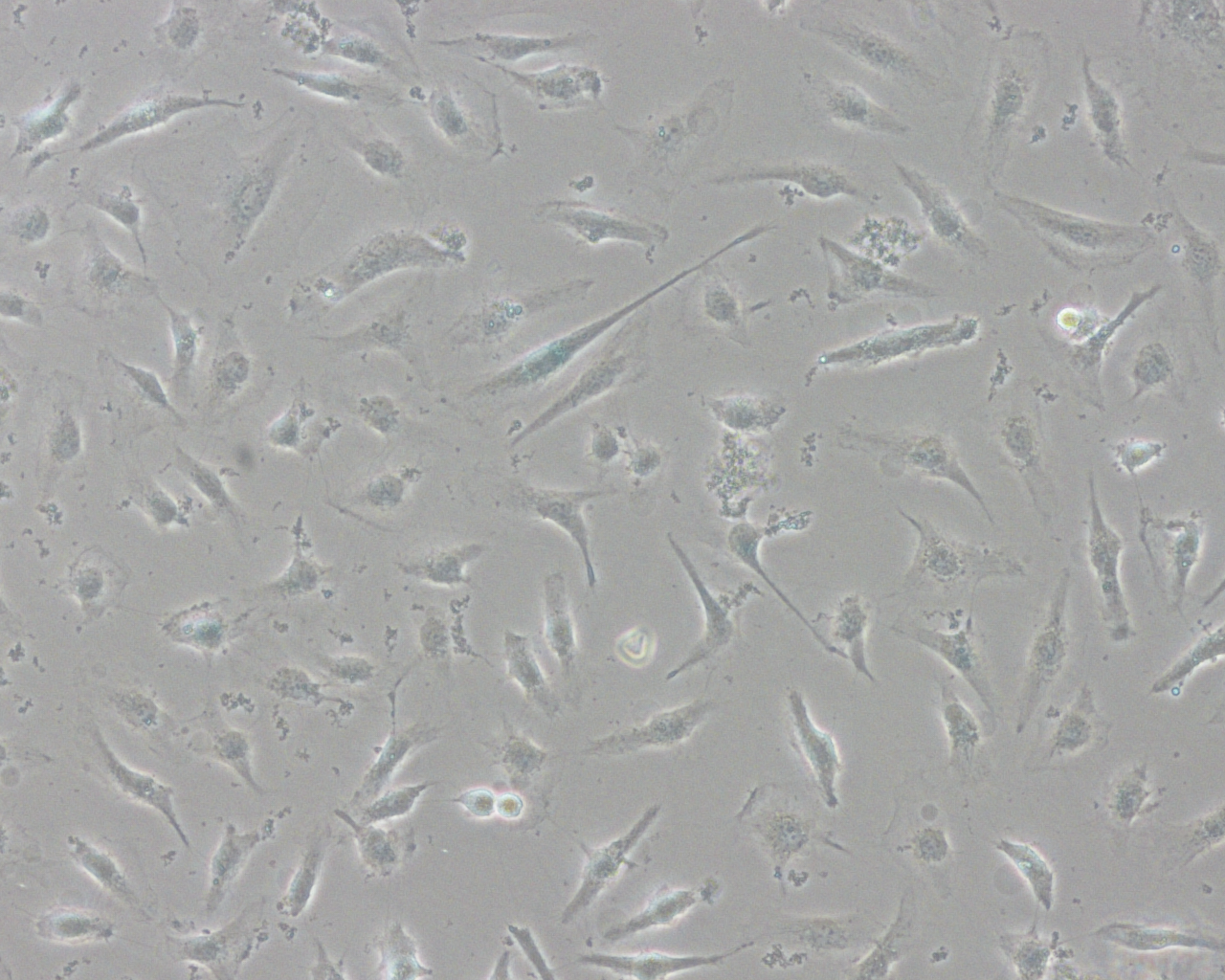

Supplement: Supplementary file 6 — Source data Fig. 4 [file 44319_2024_176_MOESM6_ESM.zip › SourceDara_ForFigure4/4K/pPAX5+pSIRT6-0Gy.tif]

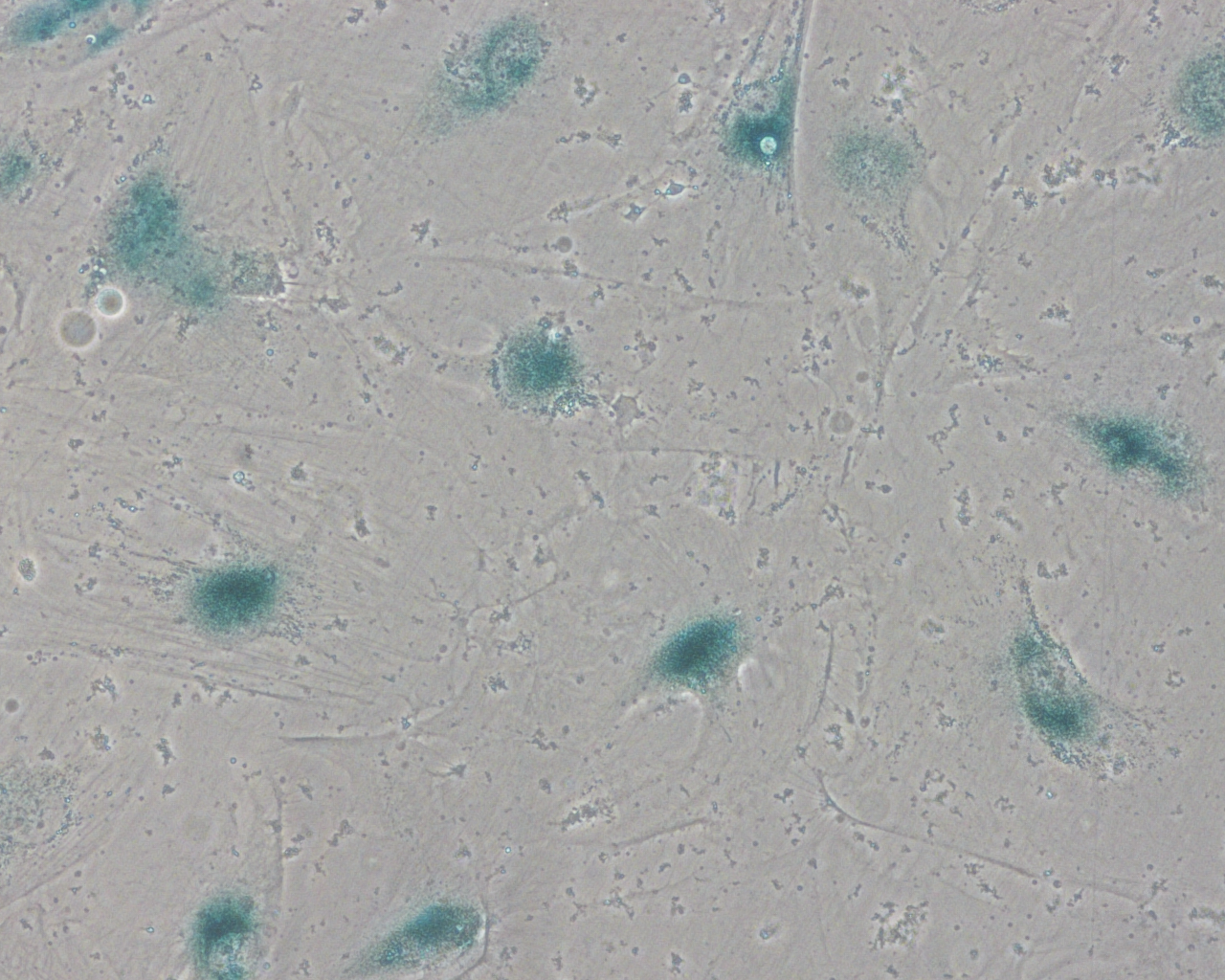

Supplement: Supplementary file 6 — Source data Fig. 4 [file 44319_2024_176_MOESM6_ESM.zip › SourceDara_ForFigure4/4K/pPAX5+pSIRT6-10Gy.tif]

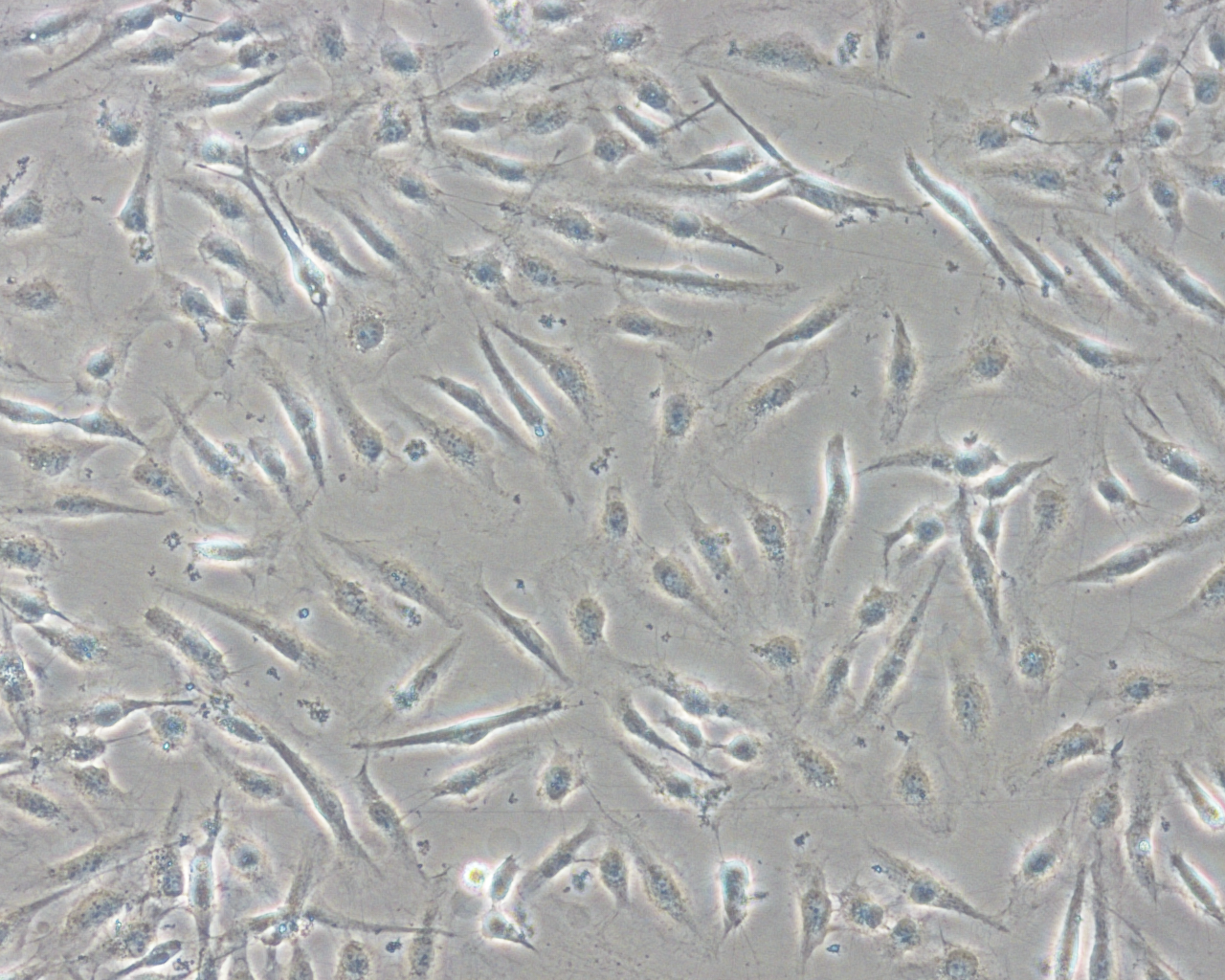

Supplement: Supplementary file 6 — Source data Fig. 4 [file 44319_2024_176_MOESM6_ESM.zip › SourceDara_ForFigure4/4K/pPAX5-0Gy.tif]

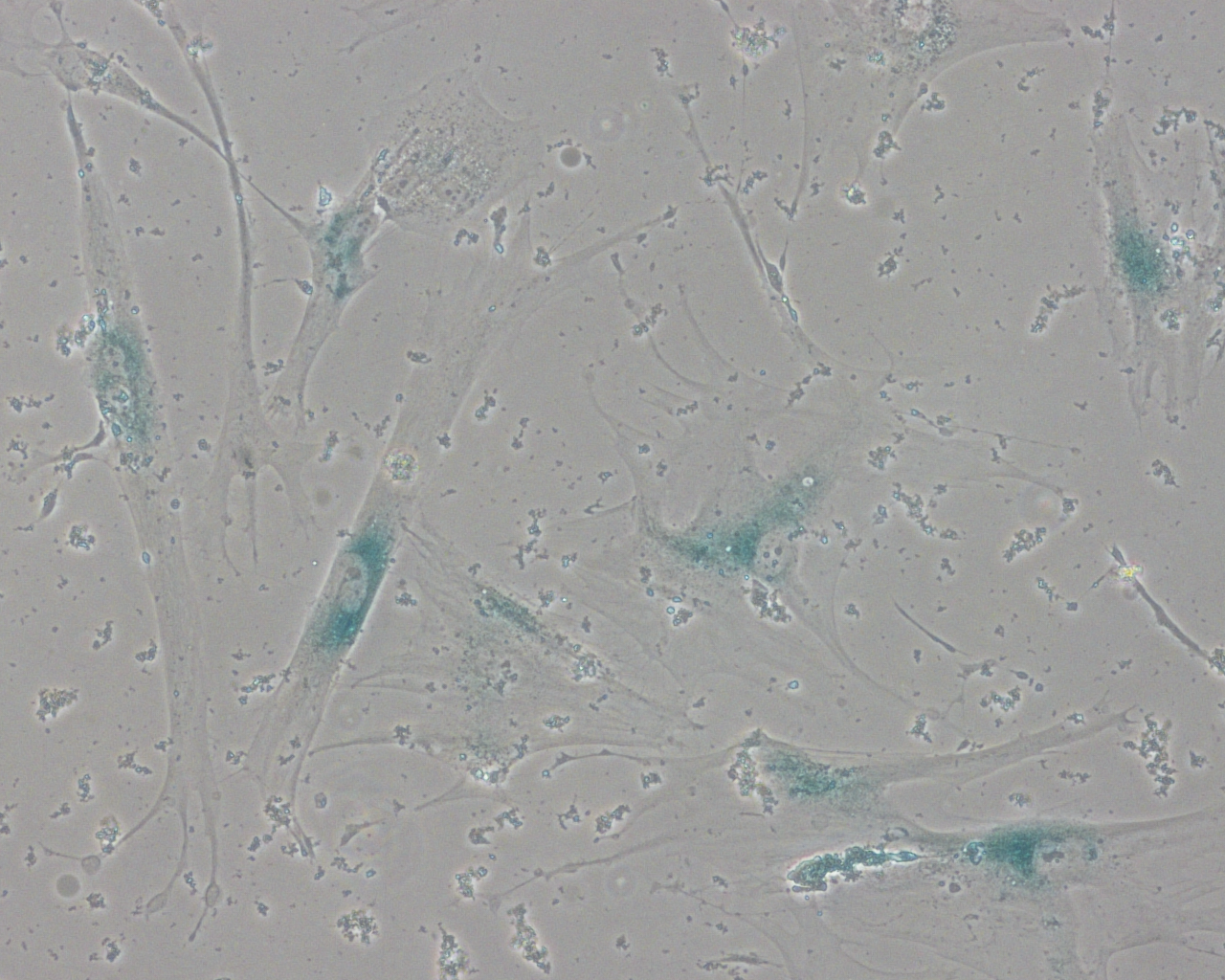

Supplement: Supplementary file 6 — Source data Fig. 4 [file 44319_2024_176_MOESM6_ESM.zip › SourceDara_ForFigure4/4K/pControl-10Gy.tif]

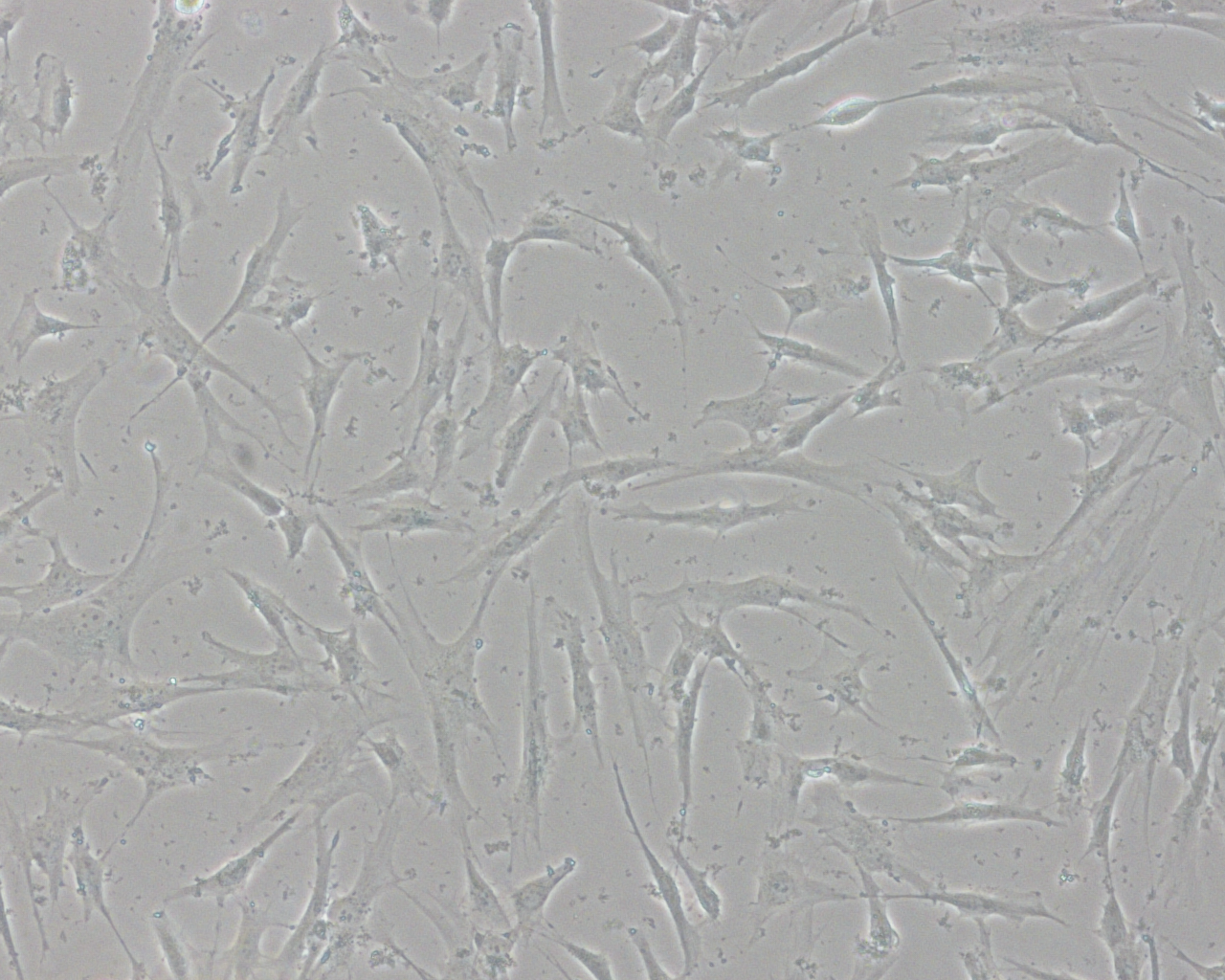

Supplement: Supplementary file 6 — Source data Fig. 4 [file 44319_2024_176_MOESM6_ESM.zip › SourceDara_ForFigure4/4K/pSIRT6-0Gy.tif]

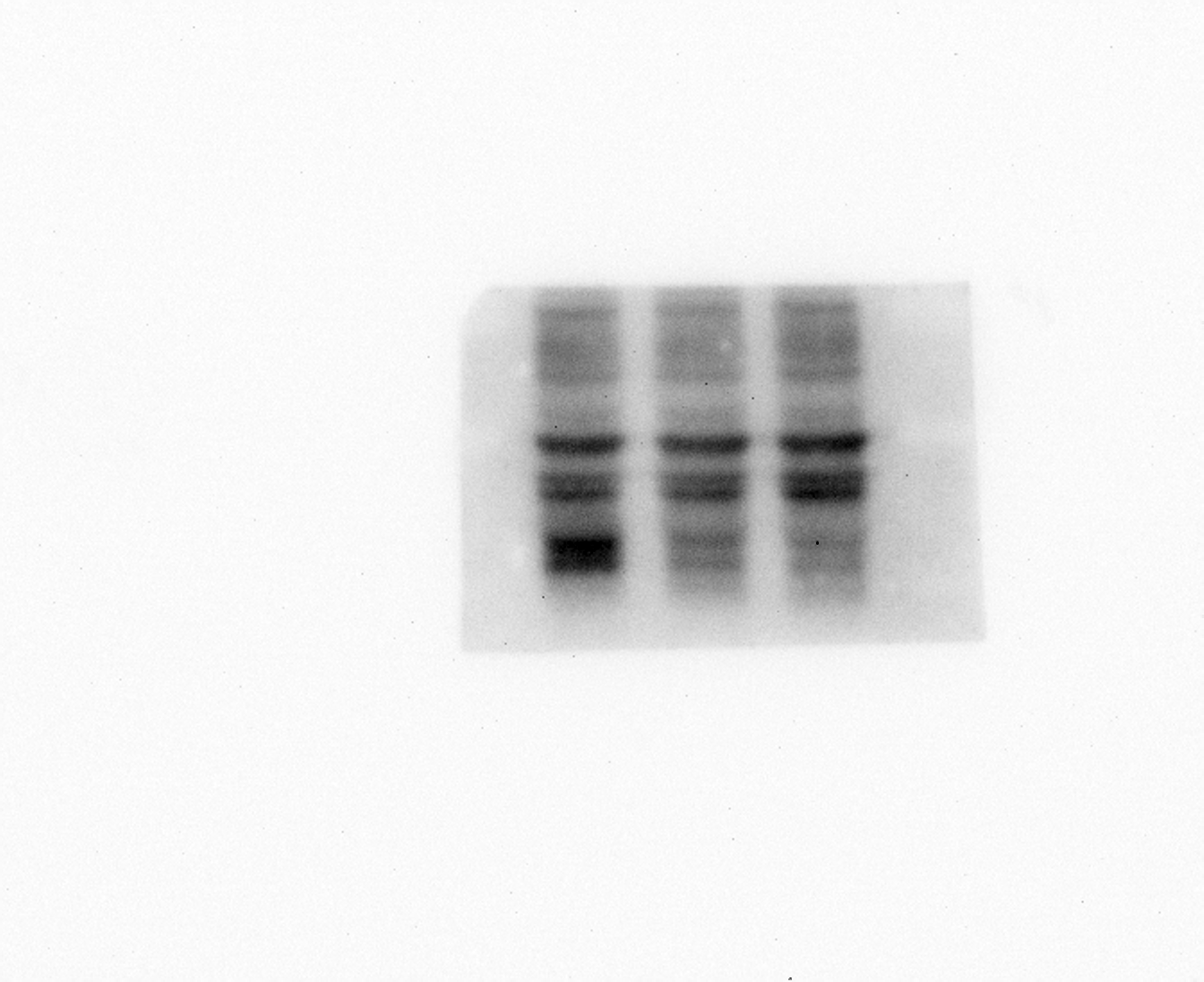

Supplement: Supplementary file 6 — Source data Fig. 4 [file 44319_2024_176_MOESM6_ESM.zip › SourceDara_ForFigure4/4A/western blot-TUBULIN.Tif]

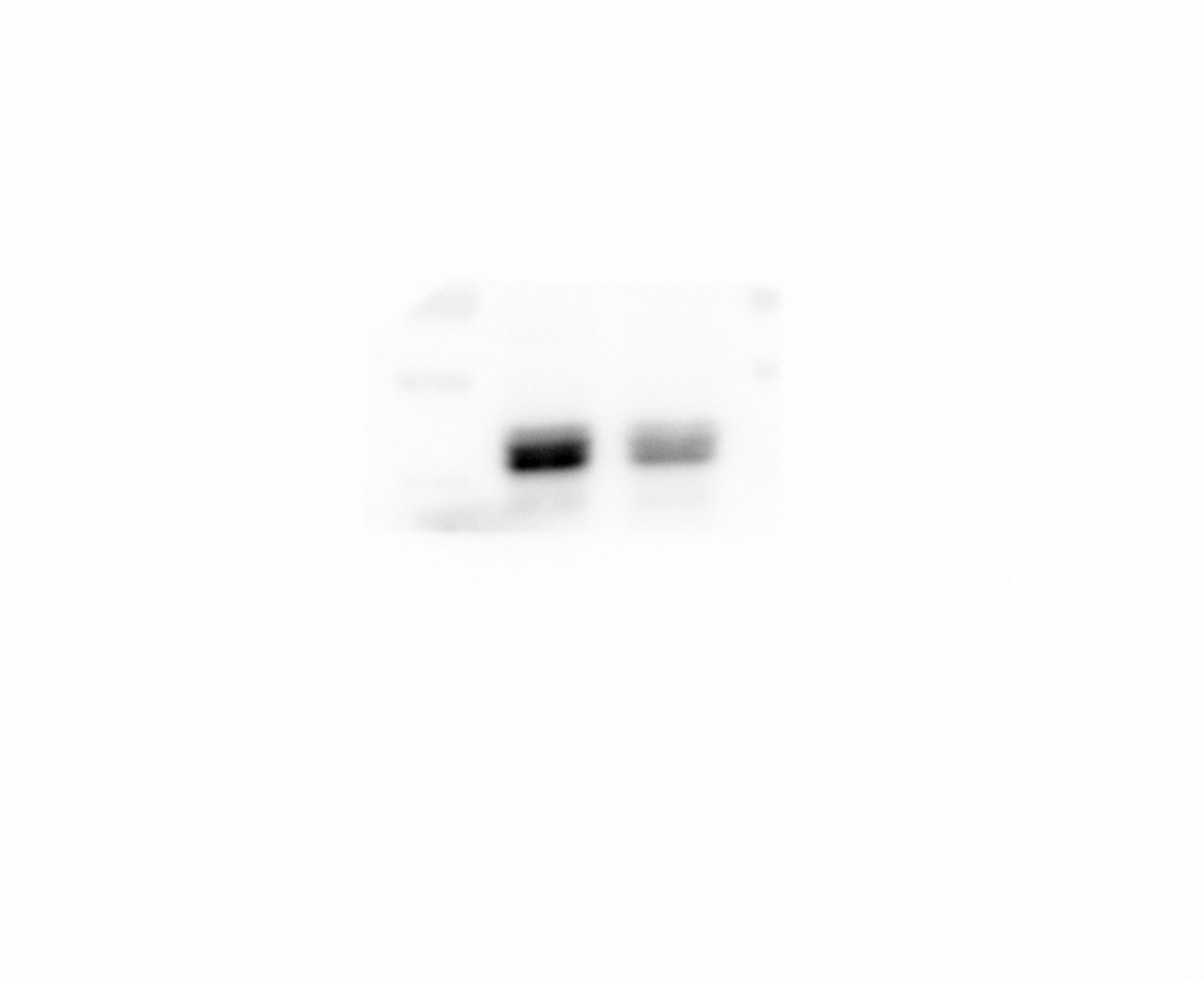

Supplement: Supplementary file 6 — Source data Fig. 4 [file 44319_2024_176_MOESM6_ESM.zip › SourceDara_ForFigure4/4A/western blot-SIRT6.Tif]

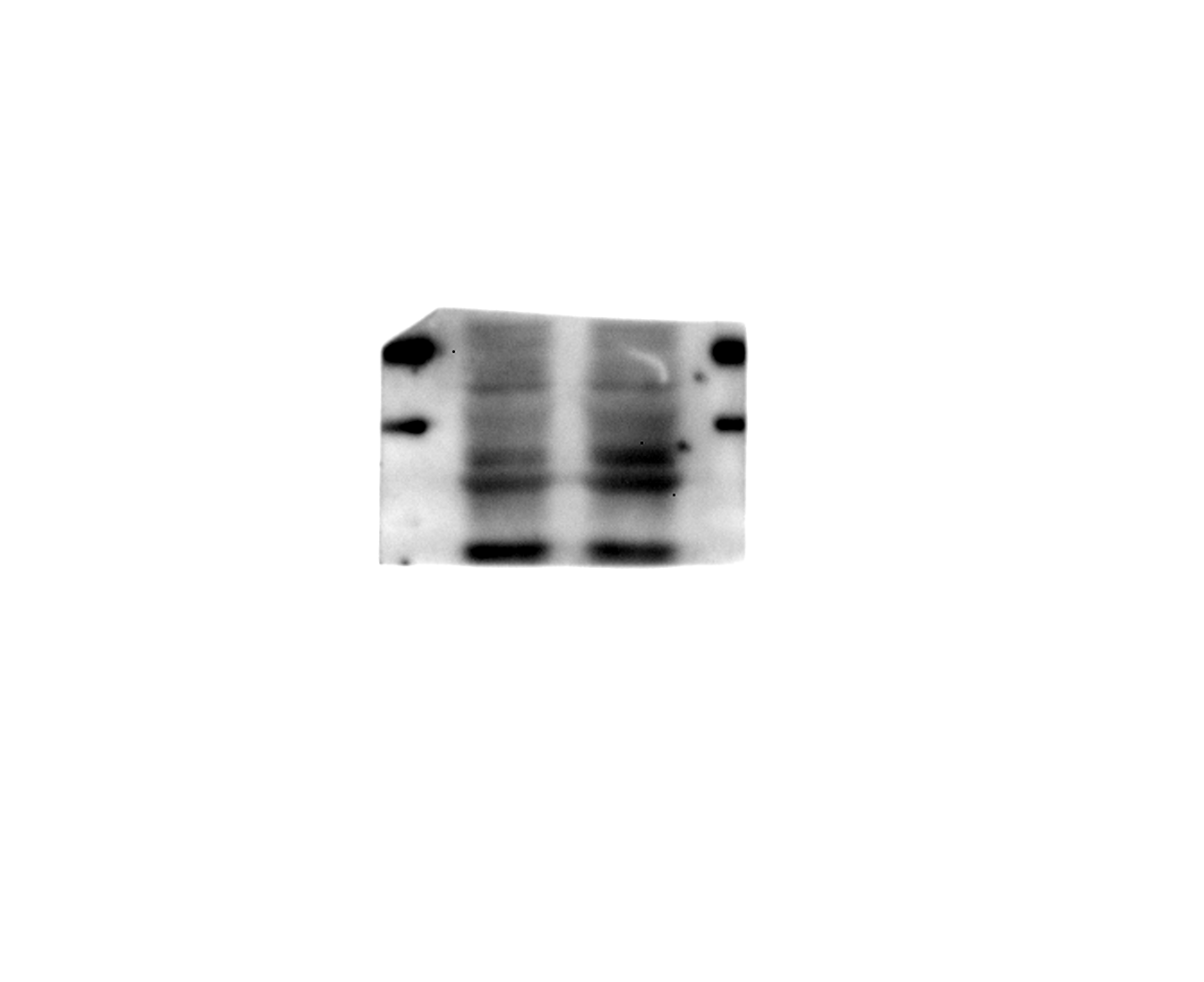

Supplement: Supplementary file 6 — Source data Fig. 4 [file 44319_2024_176_MOESM6_ESM.zip › SourceDara_ForFigure4/4A/western blot-PAX5.Tif]
