# Supplementary material for: The transcription factor PAX5 activates human LINE1 retrotransposons to induce cellular senescence
Source: EMBO Rep. 2024 Jun 12;25(8):9. doi: 10.1038/s44319-024-00176-9 (PMC11315925; doi:10.1038/s44319-024-00176-9)
Supplement: Supplementary file 7 — Expanded View Figures [file 44319_2024_176_MOESM7_ESM.pdf]

Expanded View Figure

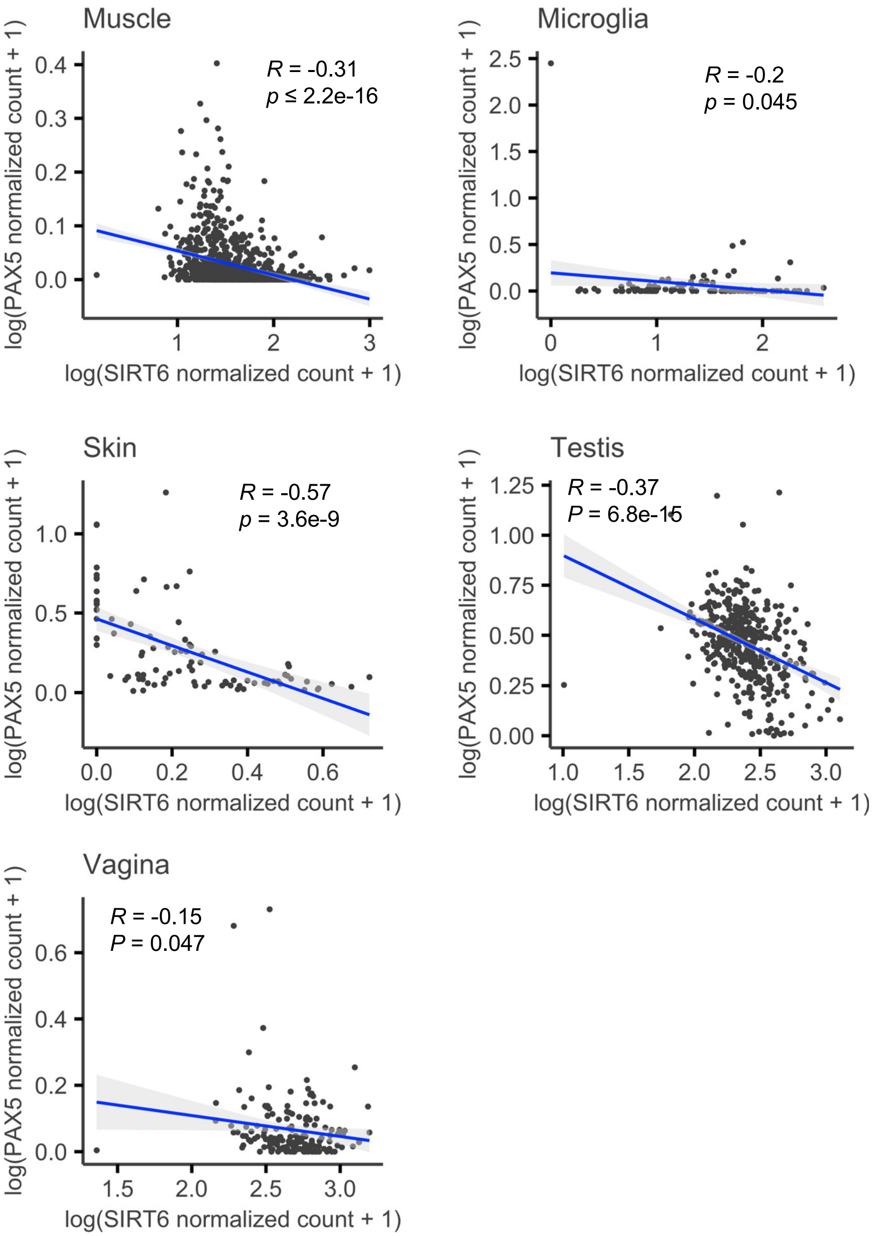

Figure EV1. SIRT6 expression is negatively correlated with PAX5 in multiple tissues.

Pearson correlation of DESeq2 normalized counts of SIRT6 and PAX5 of muscle (GTEx, $n = 881$), microglia (GSE99074, $n = 71$), skin (GSE85861, $n = 91$), testis (GTEx, $n = 410$) and vagina (GTEx, $n = 173$) tissue.

© The Author(s)
